# Lactic Acid Fermentation Improves the Bioaccessibility and Functional Quality of Reduced‐Calorie Sour Cherry Beverages During Cold Storage

**DOI:** 10.1002/fsn3.72038

**Published:** 2026-06-19

**Authors:** Perihan Kubra Akman, Gulsum Ucak‐Ozkaya, Gozde Kutlu, Fatih Tornuk, Hasan Yetim

**Affiliations:** ^1^ Faculty of Chemical and Metallurgical Engineering, Department of Food Engineering Yildiz Technical University Istanbul Türkiye; ^2^ Scientific Research Projects Coordination Unit Yildiz Technical University Istanbul Türkiye; ^3^ Faculty of Fine Arts, Design and Architecture, Department of Gastronomy and Culinary Arts Ankara Medipol University Ankara Türkiye; ^4^ Faculty of Health Sciences, Department of Nutrition and Dietetics Sivas Cumhuriyet University Sivas Türkiye; ^5^ Faculty of Engineering and Natural Sciences, Food Engineering Department Istanbul Sabahattin Zaim University Istanbul Türkiye

**Keywords:** bioactive properties, cytotoxic activity, in vitro bioaccessibility, lactic acid fermented, sour cherry

## Abstract

This study aimed to investigate the fermentation of reduced‐calorie sour cherry‐based beverages using three individual probiotic strains (*Lactiplantibacillus plantarum* SH5, *Limosilactobacillus fermentum* SH10, and *Lactiplantibacillus pentosus* SH14) and one mixed‐strain formulation, and to evaluate these beverages together with unfermented control beverages in terms of their physicochemical, microbiological, bioactive, and cytotoxic properties during 28 days of cold storage. The fermented beverages exhibited pH values of 3.54–3.63, turbidity of 0.614–0.779, total soluble solids of 24.93–29.87 °Brix, and dry matter content of 27.62%–30.75%, with color parameters recorded as L* (15.08–16.66), a* (12.80–18.21), and b* (–5.78 to −3.05). Phenolic profiling identified catechin (31.05–32.90 ppm), chlorogenic acid (10.82–11.05 ppm), and myricetin (5.05–6.90 ppm) as the predominant compounds, although most phenolics gradually declined during storage. Despite higher initial total phenolic content (TPC) in control samples, lacto‐fermented beverages exhibited superior recovery values (%), indicating enhanced bioaccessibility during in vitro digestion. Calorie reductions reached up to 46.46% in SCN9‐SH10, while viable LAB populations remained above 10^6^ CFU/mL throughout 21 days of storage (7.01–7.65 log CFU/mL). TPC ranged from 214.75 to 353.67 mg gallic acid equivalent (GAE)/100 mL, DPPH scavenging activity from 213.54 to 274.02 mg Trolox equivalent (TE)/100 mL, and cupric reducing antioxidant capacity (CUPRAC) values from 924.46 to 1576.71 mg TE/100 mL, with fermented samples consistently demonstrating higher antioxidant capacity than controls. Antibacterial activity was observed only in SCN9‐Mix and control samples against *Bacillus cereus* ATCC 11778, while cytotoxicity assays indicated modest inhibitory effects toward MCF‐7 breast cancer cells, with cell viability ranging from 87.64% to 94.45% in freshly prepared samples and decreasing over 28 days. Collectively, these findings demonstrate that lactic acid fermentation effectively enhances the functional, microbiological, and bioactive properties of reduced‐calorie sour cherry‐based beverages.

## Graphical abstract

Fermentation of reduced‐calorie sour cherry beverages with specific lactic acid bacteria reduces caloric content by up to 46% while significantly improving in vitro phenolic bioaccessibility, antioxidant capacity, and probiotic viability during 28 days of cold storage.

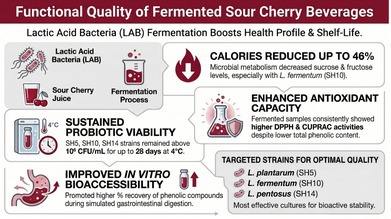

## 1 Introduction

Probiotics have been traditionally consumed through fermented dairy products; however, limitations such as lactose intolerance, cholesterol‐related concerns, and the growing consumer preference for vegetarian diets have reduced the acceptability of animal‐based probiotic foods. Therefore, there has been an increasing interest in non‐dairy alternatives in recent years, since fruit juices are considered a nutritionally valuable and sensorially appealing medium for probiotic delivery (Akman et al. 2025; Fonteles et al. 2012). At the same time, unhealthy dietary patterns remain a major contributor to obesity and related metabolic disorders, whereas regular fruit and vegetable consumption is associated with health promotion due to their rich content of minerals, vitamins, and phenolic compounds exhibiting antiproliferative, anti‐inflammatory, and antioxidant activities (Guérin et al. 2023). Within this context, sour cherry ( *Prunus cerasus* L.), a member of the Rosaceae family, represents a fruit of particular importance in Türkiye's juice industry, distinguished by its high phenolic content and strong antioxidant potential (Oksuz et al. 2019). According to the Food and Agriculture Organization report, Türkiye produces approximately 211,291 tons annually, underscoring its global significance (FAO 2023). Sour cherry juice is also one of the most widely consumed fruit juices worldwide, appreciated for its characteristic color, flavor, aroma, and health‐promoting properties. Despite being classified as a high‐acid product, its quality remains susceptible to deterioration caused by spoilage microorganisms and enzymatic activity (Akdemir Evrendilek 2024).

Lactic acid fermentation is a well‐established method for extending shelf life of foods while enhancing their nutritional quality and sensory properties, particularly fruits and vegetables, without compromising their micronutrient content (Anumudu et al. 2024; Ucak‐Ozkaya 2024). The sensory profile of fermented products is largely dependent on the choice of starter culture (Admassie 2018; Akman et al. 2025). Moreover, lactic acid fermentation has been reported to increase radical scavenging activity (Khubber et al. 2022), elevate total phenolic content (Szutowska 2020), and reduce hemolysis of blood cells under oxidative stress (Guérin et al. 2023). The successful application of lactic acid bacteria (LAB) in foods and beverages fermentation depends largely on strain selection and stability. While LAB have long been employed to improve flavor, texture, shelf life, and probiotic properties, their effectiveness is challenged by the need to select strains suitable for diverse product matrices and to ensure their stability throughout processing and storage (Anumudu et al. 2024).

Previous studies have widely demonstrated that temperature significantly affects the physicochemical properties, phenolic stability, antioxidant capacity, organic acid profile, and microbial stability of lactic acid–fermented fruit beverages during storage (Erol et al. 2024; Kwaw, Tchabo, et al. 2018; Güler and Tokuşoğlu 2024; Akman et al. 2025). However, in almost all of these studies, temperature was considered only as a post‐production storage parameter, while the fermentation process itself was carried out under conventional room or optimal LAB growth temperatures. This distinction is critical because fermentation temperature directly governs LAB metabolic pathways. Under cold fermentation conditions, reduced enzymatic activity, slower carbohydrate metabolism, and altered stress‐response mechanisms promote different carbon utilization patterns and organic acid formation compared to room‐temperature fermentation (Cirlini et al. 2020). As a result, beverages fermented directly under cold conditions may develop substantially different sugar consumption patterns, acid profiles, bioactive stability, and physicochemical characteristics than those fermented at room temperature and later stored under refrigeration (Akman et al. 2025). Despite the well‐established influence of storage temperature, the characterization of beverages fermented directly under cold conditions remains largely unexplored in the literature. Therefore, evaluating cold‐fermented beverages is essential to understand how fermentation temperature itself shapes the final beverage matrix, rather than merely assessing temperature effects after production.

Sour cherries are especially rich in polyphenols, which exert antioxidant, antidiabetic, antiobesity, antimutagenic, and anticarcinogenic effects, thereby contributing to their health‐promoting potential. The physiological functionality of these compounds depends on their bioaccessibility within the gastrointestinal tract, which is commonly assessed using in vitro digestion models due to their practicality, cost‐effectiveness, and avoidance of the ethical limitations inherent in human trials (Ozkan et al. 2025). Ultimately, the bioaccessibility of phenolic compounds—defined by their absorption and distribution to target tissues—determines their health benefits and is influenced by multiple factors, including food composition, genotype, matrix characteristics, gastric conditions (*e.g.,* pH, redox potential, health status), intestinal absorption, as well as individual nutritional and physiological variations (Oksuz et al. 2019).

Previous studies have explored the fermentation of sour cherry juice under various conditions, including the assessment of its probiotic potential with multiple *Lactobacillus* strains (Perjéssy et al. 2020), optimization of fermentation parameters for spirit production (Pham et al. 2021), and characterization of volatile profiles following fermentation with *L. rhamnosus* (Gao et al. 2022). Other investigations have examined physicochemical, antioxidant, and sensory characteristics of fermented cherry beverages (Rios‐Corripio and Guerrero‐Beltrán 2020), the use of dairy‐ and plant‐derived lactobacilli as starter cultures (Ricci et al. 2019), and aroma profiles of sweet cherry juice fermented by lactic acid bacteria (Wang et al. 2023). However, to the best of our knowledge, no research has addressed the development of lactic acid–fermented reduced‐calorie sour cherry beverages with comprehensive evaluation of their physicochemical, bioactive, antimicrobial, and cytotoxic properties together with in vitro gastrointestinal bioaccessibility during cold storage. Therefore, the aim of the present study was to produce such beverages using *Lactiplantibacillus plantarum* SH5, *Limosilactobacillus fermentum* SH10, and *Lactiplantibacillus pentosus* SH14.

## 2 Materials and Methods

### 2.1 Materials

Sour cherry juice concentrates ( *Prunus cerasus* L.) in 250 g aseptic packages were obtained from Goknur Gıda A.S. (Kayseri, Türkiye). The probiotic strains *Limosilactobacillus fermentum* SH10, *Lactiplantibacillus pentosus* SH14, and *Lactiplantibacillus plantarum* SH5, sourced from Yildiz Technical University, Department of Food Engineering (Istanbul, Türkiye), have been previously characterized for their probiotic potential, including tolerance to osmotic stress (Akman et al. 2021; Bagdat, Kutlu, and Tornuk 2024).

### 2.2 Selection of the Sour Cherry Based Lacto‐Fermented Beverages for Cold Storage

In this study, the development and characterization of lactofermented sour cherry‐based beverages were carried out through a three‐stage selection process as previously mentioned by Akman et al. (2025) for apricot beverages. In the first stage, fermentation optimization was performed using three probiotic strains (*Lactiplantibacillus plantarum* SH5, *Limosilactobacillus fermentum* SH10, and *Lactiplantibacillus pentosus* SH14) under different conditions. Beverages were prepared by adding sucrose at 9%, 12%, or 15% (w/v) in accordance with Turkish Food Codex (OG 2014), and fermentation was conducted at 20°C, 28.5°C, or 37°C for 20 h. A total of 27 samples were monitored for LAB growth at 4‐h intervals up to 48 h, and freshly fermented samples were evaluated for sensory acceptability. Subsequently, a 10‐day pre‐storage at 4°C was applied to assess the potential probiotic viability. Strains showing the highest bacterial counts, stable viability, and favorable sensory scores were identified as optimal. In the second stage, caloric content was considered to select reduced‐calorie formulations while maintaining probiotic viability and sensory quality. Based on these criteria, one representative beverage was chosen for each strain. These parameters were integrated using a weighted scoring system [Total score = (*A* × 0.4) + (*B* × 0.2) + (*C* × 0.4)], where *A* represents LAB counts at the end of fermentation, *B* represents LAB counts after 10 days of storage, and *C* represents sensory acceptance. Finally, in the third stage, three reduced‐calorie sour cherry formulations containing individual probiotic strains (SCN9‐SH10, SCN12‐SH5, SCN12‐SH14) and one mixed‐strain formulation (SCN9‐Mix), along with unfermented control samples (SCN9‐Control, SCN12‐Control), were selected for detailed physicochemical, microbiological, bioactive, and cytotoxic characterization during cold storage.

### 2.3 Preparation and Lacto‐Fermentation of Sour Cherry‐Based Beverages

The fruit content of the sour cherry‐based beverages was standardized to 30% (w/v) by dilution with potable water. Sucrose was subsequently added at concentrations of 9%, 12%, or 15% (w/v). The formulations were pasteurized at 90°C for 30 s using a laboratory‐scale plate heat exchanger (ASH10a Laval, model T2‐BFG, Sweden) and aseptically filled into sterile amber glass bottles as previously reported by Akman et al. (2025).

The selected starter cultures—*Lactiplantibacillus plantarum* SH5, *Limosilactobacillus fermentum* SH10, and *Lactiplantibacillus pentosus* SH14—were subjected to double activation in MRS broth at 37°C for 24 h. Following activation, cultures were centrifuged, washed, and resuspended in sterile peptone water to one‐quarter of the original volume. Culture concentrations were adjusted spectrophotometrically based on absorbance measurements at 590 nm (Pereira et al. 2011). The suspensions were standardized to an initial inoculation level of approximately 9 log CFU/mL and aseptically inoculated into the pasteurized sour cherry‐based beverages at 4% (v/v), corresponding to ~10^9^ CFU/mL. Fermentation was carried out under static conditions (without agitation) for 20 h at the previously optimized temperature for each strain. At the end of fermentation, day‐0 samples were collected for analysis, and the remaining bottles were stored at 4°C for 28 days. After fermentation at optimized conditions, the beverages—SCN9‐SH10 (9% sucrose, *Limosilactobacillus fermentum* SH10), SCN12‐SH5 (12% sucrose, *Lactiplantibacillus plantarum* SH5), SCN12‐SH14 (12% sucrose, *Lactiplantibacillus pentosus* SH14), and SCN9‐Mix (9% sucrose, mixed culture of SH5, SH10, and SH14)—were stored at 4°C, and their physicochemical, microbiological, bioactive, and cytotoxic properties were systematically monitored throughout the storage period for all subsequent analyses in the study.

Bacterial cultures were subjected to double activation to ensure sufficient population growth, which was verified by measuring absorbance at 590 nm using a spectrophotometer (Pereira et al. 2011). LAB cells were harvested by centrifugation from MRS broth and resuspended in sterile peptone water at one‐quarter of the original broth volume. The resulting suspensions were aseptically inoculated into 100 mL glass bottles containing prepared sour cherry juice at a final concentration of 4% (v/v). The inoculated juices were fermented for 20 h, after which samples designated for day‐0 analyses were collected, and the remaining samples were stored at 4°C for 28 days. Unfermented sour cherry juice served as a control.

### 2.4 Determination of Total LAB Counts

The total LAB counts in the lactic acid fermented samples were determined using the spread plate method. Briefly, 10 mL of the sample was mixed with 90 mL of sterile peptone water, and serial dilutions were prepared. Appropriate dilutions were plated on MRS agar and incubated at 35°C for 48 h under anaerobic conditions. The results were expressed as log CFU/mL (de Man et al. 1960). LAB counts were determined at regular intervals throughout the storage period.

### 2.5 Physicochemical Properties

The pH of the samples was measured by immersing the probe of a pH meter—previously calibrated with standard buffer solutions—directly into the samples equilibrated to 20°C ± 2°C (Akman et al. 2025). Titratable acidity was determined by titrating the samples with 0.02 N NaOH to an endpoint of pH 8.2, and the results were expressed as lactic acid equivalents (%) (Yoon et al. 2005). The total solid content of the lactofermented sour cherry‐based fruit juices was quantified gravimetrically by drying the samples in a vacuum oven at 70°C (AOAC 1990). The total soluble solids of the reduced‐calorie beverage samples were determined at 20°C using a handheld refractometer (Bagdat, Kutlu, and Tornuk 2024). Color properties of the fermented sour cherry juices were analyzed using a Minolta colorimeter (CR‐400, Konica Minolta Sensing, Osaka, Japan), where L* (lightness), a* (red/green), and b* (yellow/blue) parameters were recorded, and the total color difference (ΔE*) was calculated (Akman et al. 2025). Turbidity of the lactofermented sour cherry‐based beverages (diluted 1:10 with distilled water) was assessed by measuring absorbance at 540 nm using a UV–Vis spectrophotometer (Shimadzu UV‐1800) (Akman et al. 2025).

### 2.6 Determination of Bioactive Properties and Antioxidant Activity

The total phenolic content (TPC) of the samples was determined using the Folin–Ciocalteu method. Briefly, 0.2 mL of sample extract was mixed with 1.5 mL of 10% (v/v) Folin–Ciocalteu reagent and incubated for 5 min at room temperature. Then, 1.5 mL of 7.5% (w/v) sodium carbonate solution was added, and the mixture was incubated in the dark for 30 min at room temperature. Absorbance was measured at 765 nm using a UV–Vis spectrophotometer (Shimadzu UV‐1800). The results were calculated from a gallic acid calibration curve (0–200 mg/L, *R* ^2^ > 0.99) and expressed as mg gallic acid equivalents (GAE) per 100 mL of sample (Erol et al. 2024).

The DPPH radical scavenging activity was determined by mixing 0.1 mL of the sample with 4.9 mL of 0.1 mM ethanolic DPPH solution. The mixture was vortexed and incubated in the dark at room temperature for 20 min. Absorbance was recorded at 517 nm against ethanol as a blank using a UV–Vis spectrophotometer. Results were expressed as mg TE/100 mL, based on a standard calibration curve with a linear range of 0.05–0.5 mg mL^−1^ (*R* ^2^ = 0.996) (Erol et al. 2024).

The total antioxidant capacity was measured using the CUPRAC method, based on the reduction of Cu(II) to Cu(I) ions. In this method, 1 mL of 0.01 M CuCl_2_ solution, 1 mL of 0.0075 M neocuproine ethanolic solution, and 1 mL of 1 M ammonium acetate buffer (pH 7.0) were transferred into a test tube. Subsequently, 0.1 mL of the sample and 1 mL of distilled water were added to the mixture. The reaction mixture was vortexed and incubated at room temperature for 60 min. Absorbance was measured at 450 nm against a reagent blank. Results were expressed as mg TE/100 mL, based on a standard calibration curve with a linear range of 0.05–1.0 mg mL^−1^ (*R* ^2^ = 0.991) (Apak et al. 2004).

### 2.7 Determination of Sucrose and Fructose Content

The sugar content in lactic acid fermented fruit‐based beverages was analyzed using an HPLC system (Agilent 1100, Agilent Technologies, Palo Alto, USA) equipped with a refractive index detector (RID) and an Agilent Zorbax carbohydrate column (5 μm, 4.6 mm × 150 mm). Liquid samples were filtered through a 0.45 μm membrane filter prior to injection. The analysis conditions were as follows: mobile phase—80% acetonitrile and 20% water; flow rate—1.4 mL/min; injection volume—20 μL; column temperature—25°C. Sucrose and fructose contents were quantified based on retention times using calibration curves prepared from sugar standards (Karaman et al. 2017).

### 2.8 Determination of Antimicrobial Activity

The antimicrobial activity of the sour cherry‐based beverages was determined using the agar well diffusion method. The test microorganisms included *Staphylococcus aureus, Escherichia coli* O157:H7, *Bacillus cereus, Listeria monocytogenes, Candida albicans*, and *Saccharomyces cerevisiae* . Culture media (Nutrient Agar for bacteria and Dichloran Rose Bengal Chloramphenicol Agar (DRBC) for yeasts) were inoculated with 1% (v/v) of the activated test microorganism, poured into Petri dishes, and allowed to solidify. Wells were then punched into the agar, and 50 μL of the beverage samples were inoculated into each well. To evaluate the effect of pH on inhibition, the pH values of the lacto‐fermented sour cherry‐based beverages were adjusted to 5, 6, and 7 using 0.1 N NaOH, and the results were compared. Petri dishes were incubated at 37°C for bacteria and 28°C for yeasts for 24–48 h, after which the inhibition zones around the wells were measured. Results were expressed in millimeters. Unfermented fruit nectar was used as a negative control in these tests (Česonienė et al. 2012).

### 2.9 Determination of LAB Counts

During fermentation optimization, LAB enumeration was conducted using the spread plate method at 4‐h intervals. Appropriate serial dilutions were prepared in sterile saline solution, plated on MRS agar, and incubated under anaerobic conditions at 35°C for 48 h (de Man et al. 1960).

### 2.10 Determination of Total Caloric Content

The proximate composition of the beverages was determined using standard AOAC (2000) methods. Moisture content was measured by the hot air oven method, while ash, protein, fat, and crude fiber contents were analyzed according to the respective AOAC procedures. Carbohydrate content was calculated by difference using the following equation:

(1) $$\text{Carbohydrate}\;\left(\%\right)=100-\left[\text{Moisture}\;\left(\%\right)+\mathrm{Ash}\;\left(\%\right)+\text{Protein}\;\left(\%\right)+\mathrm{Fat}\;\left(\%\right)+\text{Crude fiber}\;\left(\%\right)\right]$$

The total caloric value of the beverages was calculated using the Atwater conversion factors as follows:

(2) $$\text{Total caloric value}\;\left(\text{kcal}/100\;\mathrm{mL}\right)=\left(\text{Protein}\times 4\right)+\left(\mathrm{Fat}\times 9\right)+\left(\text{Carbohydrate}\times 4\right)$$

Results were expressed as kcal per 100 mL (Ellefson 1993).

### 2.11 In Vitro Cytotoxic Activity

The cytotoxicity of sour cherry‐based beverages was assessed on the human breast cancer cell line MCF‐7 using the XTT assay. Cells stored in liquid nitrogen were thawed at 37°C, washed by centrifugation in DMEM‐F12 medium supplemented with 10% PBS, and cultured in 25 cm^2^ flasks under standard conditions until confluency was reached. After nine days of culture, including medium renewals on Days 2 and 7, cells were trypsinized, counted with a hemocytometer, and seeded into 96‐well plates at a density of 1 × 10^4^ cells/well. Following 24 h attachment at 37°C (5% CO_2_), cells were exposed to varying concentrations of the sour cherry‐based beverages and incubated for another 24 h. Subsequently, the medium was replaced with XTT reagent (0.5 mg/mL), and plates were incubated for 3 h. Cell viability was quantified spectrophotometrically at 450 nm, with the percentage of viable cells calculated relative to untreated controls, as described by Kutlu et al. (2024).

(3) $$\text{Cell viability}\;\left(\%\right)=\frac{{\mathrm{OD}}_{\text{Sample}}}{{\mathrm{OD}}_{\text{Control}}}\times 100$$

### 2.12 Determination of the In Vitro Bioaccessibility of the Bioactive Properties of Sour Cherry‐Based Beverages

The in vitro bioaccessibility of bioactive compounds and antioxidant activity in sour cherry‐based beverages was investigated through the gastrointestinal digestion model of McDougall et al. (2005), simulating oral, gastric, and intestinal phases. In the oral stage, 2.5 mL of each sample was diluted to 20 mL with distilled water to mimic mechanical digestion. Gastric conditions were created by adding 1.5 mL pepsin solution (40 mg/mL in 0.1 M HCl), adjusting the pH to 2.0 with 5 M HCl, and incubating at 37°C in a shaking water bath (100 rpm) for 2 h; the supernatant obtained was designated as the post‐gastric fraction (P_g_). For intestinal digestion, 4.5 mL of a pancreatin (4 mg/mL) and bile salt (25 mg/mL) mixture was introduced, and titratable acidity was neutralized with 0.5 M NaHCO_3_ inside a 15 cm cellulose dialysis tube. The sealed beaker was then incubated at 37°C and 100 rpm for 2 h. The dialyzable fraction within the tube represented the intestinal‐available fraction (IN), while the residual solution outside was considered the nondialyzable fraction (OUT). All fractions (*P*_g_, IN, OUT) were stored at −20°C until analyses of total phenolic content, total flavonoid content, and antioxidant capacity were conducted, with unfermented beverages serving as control samples (Erol and Kutlu 2025).

### 2.13 Statistical Analysis

Data obtained from the laboratory experiments were processed using Microsoft Excel to calculate means and standard deviations. All analyses were conducted in triplicate. The results obtained for each sample were compiled and subjected to statistical evaluation. A two‐way analysis of variance (ANOVA) was conducted to assess the effects of formulation and storage time, and their interaction, on the measured parameters. ANOVA was performed using the Windows‐based SAS software package (SAS 8.2, North Carolina, USA). When significant differences were detected, Tukey's multiple comparison test was used to compare the means at a 95% confidence level (*p* < 0.05).

## 3 Results and Discussion

### 3.1 Optimization and Selection of the Lacto‐Fermented Sour Cherry Beverages

**FIGURE 1:**
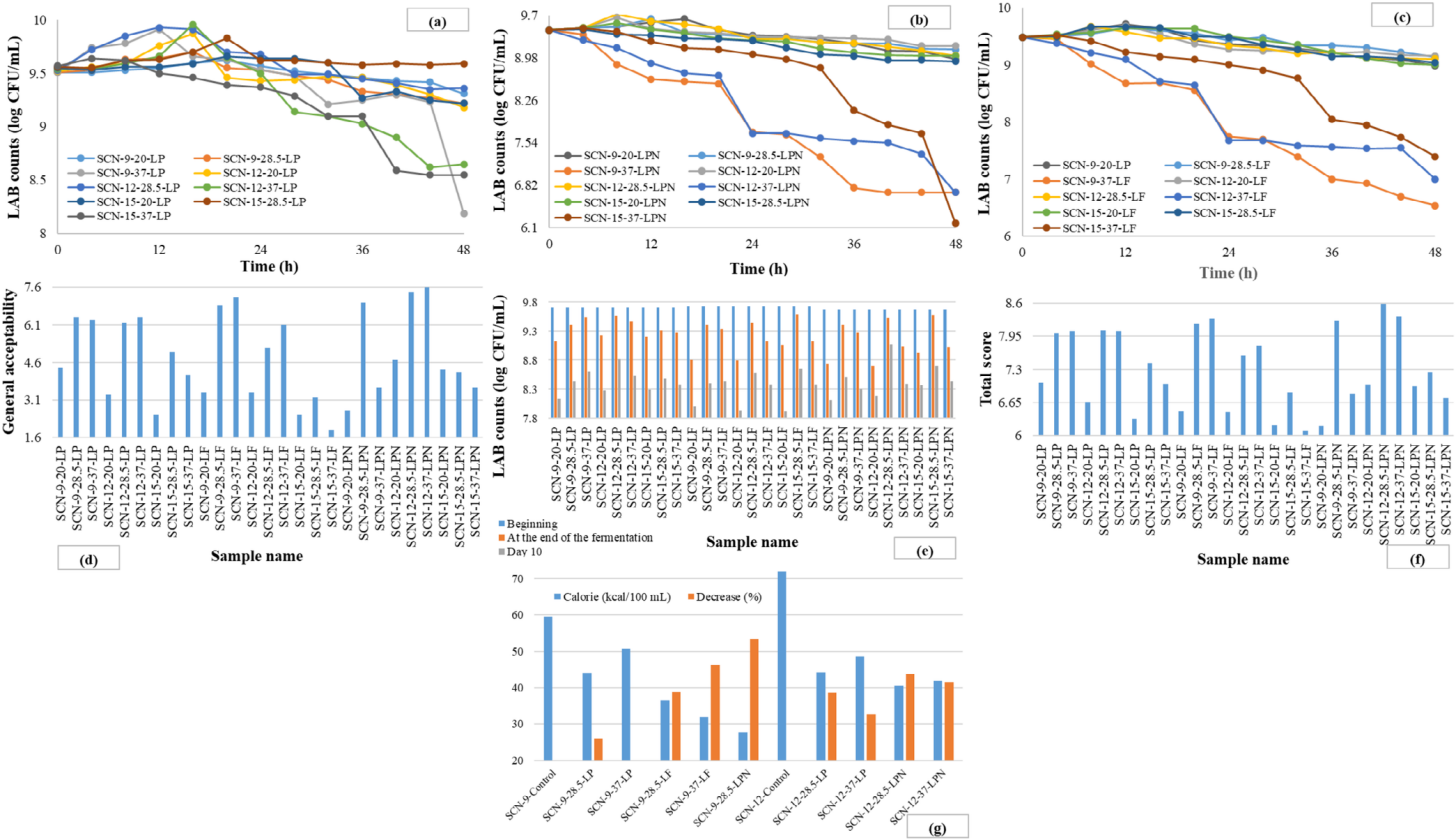
(a) LAB counts of sour cherry–based beverages fermented with *Lactiplantibacillus plantarum* SH5 (LP), (b) LAB counts of sour cherry–based beverages fermented with *Lactiplantibacillus pentosus* SH14 (LPN), (c) LAB counts of sour cherry–based beverages fermented with *Limosilactobacillus fermentum* SH10 (LF), (d) mean overall acceptability scores of probiotic sour cherry–based beverage samples, (e) LAB counts (log CFU/mL) of probiotic sour cherry–based beverages at initial, end of fermentation, and Day 10, (f) total scores calculated based on LAB counts and sensory properties of probiotic sour cherry–based beverages, and (g) total calorie values and percentage reduction rates of fermented sour cherry nectars (SCN). * Experiments were conducted under varying sugar concentrations (9%, 12%, and 15%) and fermentation temperatures (20°C, 28.5°C, and 37°C). SCN, Sour cherry nectar; SCN12‐SH14, SCN containing 12% sucrose inoculated with *Lactiplantibacillus pentosus* SH14; SCN12‐SH5, SCN containing 12% sucrose inoculated with *Lactiplantibacillus plantarum* SH5; SCN9‐Control and SCN12‐Control, Noninoculated SCN samples containing 9% and 12% sucrose, respectively; SCN9‐Mix, SCN containing 9% sucrose inoculated with a mixed culture of SH5, SH10, and SH14; SCN9‐SH10, SCN containing 9% sucrose inoculated with *Limosilactobacillus fermentum* SH10.

During optimization, the selected storage samples exhibited the highest bacterial growth with SH5, SH10, and SH14 isolates at 28.5°C and 37°C, reaching maximum counts of 9.46–9.96, 8.69–9.65, and 8.58–9.64 log CFU/mL at 16 h before entering the stationary phase (Figure 1a–c). Sensory evaluation showed that samples containing 9%–12% sugar fermented at these temperatures received higher overall acceptability scores compared to other formulations (Figure 1d). After 10 days of storage at 4°C, LAB viability was maintained at 7.92–9.07 log CFU/mL, remaining close to the probiotic threshold of 8 log CFU/mL (Figure 1e). Moreover, total scores calculated based on LAB counts and sensory properties of probiotic sour cherry–based beverages were given in Figure 1f. In terms of nutritional value, fermentation resulted in a 14.79%–53.21% calorie reduction, with the targeted 30% decrease achieved in 7 out of 9 samples (Figure 1g). These outcomes guided the selection of formulations for subsequent storage trials, ensuring a balance between microbial viability, consumer acceptance, and reduced caloric content.

### 3.2 Total Soluble Solid Content

The soluble solid content of sour cherry‐based lactic acid fermented beverages remained remarkably stable throughout the 28‐day storage period (Table 1). No meaningful changes were observed over time in either control or fermented samples, indicating limited microbial metabolic activity under refrigeration conditions. However, clear differences were evident between control and fermented beverages from the beginning of storage. Freshly prepared nectars showed °Brix values ranging from 25.33 to 29.87. Samples fermented with SH10, SH5, and SH14 exhibited significantly lower °Brix values than their respective control samples (*p* < 0.05), reflecting sugar utilization during the fermentation process rather than during storage.

**TABLE 1:** Physicochemical characteristics of lactic acid fermented sour cherry beverages during 28 days of storage, assessed at 7‐day intervals.

Analysis	Sample name	Day 0	Day 7	Day 14	Day 21	Day 28
Total soluble solids (°Brix)	SCN9‐Control	28.71 ± 0.06^Ab^	28.64 ± 0.21^Ab^	28.77 ± 0.02^Ab^	28.83 ± 0.11^Ab^	28.76 ± 0.03^Ab^
	SCN12‐Control	29.87 ± 0.06^Aa^	29.80 ± 0.10^Aa^	29.77 ± 0.06^Aa^	29.67 ± 0.06^Aa^	29.67 ± 0.15^Aa^
	SCN9‐SH10	25.33 ± 0.06^Af^	25.13 ± 0.06^ABd^	25.10 ± 0.10^ABd^	25.07 ± 0.15^ABe^	24.93 ± 0.12^Bd^
	SCN12‐SH5	27.20 ± 0.06^Ae^	27.17 ± 0.31^Ac^	27.27 ± 0.42^Ac^	27.17 ± 0.25^Ad^	27.00 ± 0.26^Ac^
	SCN12‐SH14	27.83 ± 0.06^Ad^	27.53 ± 0.25^Ac^	27.70 ± 0.20^Ac^	27.60 ± 0.17^Ac^	27.43 ± 0.15^Ac^
	SCN9‐Mix	28.55 ± 0.06^Ac^	28.69 ± 0.15^Ab^	28.41 ± 0.31^Ab^	28.45 ± 0.06^Ab^	28.52 ± 0.42^Ab^
pH	SCN9‐Control	3.54 ± 0.01^Ab^	3.54 ± 0.01^Ac^	3.54 ± 0.00^Ab^	3.54 ± 0.01^Ab^	3.54 ± 0.01^Ac^
	SCN12‐Control	3.54 ± 0.01^Ab^	3.54 ± 0.01^Ac^	3.54 ± 0.00^Ab^	3.54 ± 0.01^Ab^	3.54 ± 0.01^Ac^
	SCN9‐SH10	3.61 ± 0.01^Aba^	3.61 ± 0.01^Aab^	3.60 ± 0.01^ABCa^	3.59 ± 0.01^BCa^	3.59 ± 0.01^Cb^
	SCN12‐SH5	3.62 ± 0.01^Aa^	3.62 ± 0.00^ABab^	3.61 ± 0.01^BCa^	3.59 ± 0.01^CDa^	3.59 ± 0.01^Db^
	SCN12‐SH14	3.63 ± 0.01^Aa^	3.62 ± 0.01^Aba^	3.61 ± 0.01^BCa^	3.60 ± 0.01^Ca^	3.60 ± 0.01^Cab^
	SCN9‐Mix	3.62 ± 0.01^Aa^	3.61 ± 0.01^Bb^	3.61 ± 0.01^Ba^	3.61 ± 0.01^Ba^	3.61 ± 0.01^Ba^
Dry matter (%)	SCN9‐Control	29.84 ± 0.06^Aab^	29.81 ± 0.15^Aa^	29.90 ± 0.06^Aa^	29.86 ± 0.21^Aa^	29.81 ± 0.11^Aa^
	SCN12‐Control	30.75 ± 0.05^Aa^	30.04 ± 0.24^Ba^	29.92 ± 0.34^Ba^	29.90 ± 0.34^Ba^	29.92 ± 0.04^Ba^
	SCN9‐SH10	27.84 ± 0.18^Ac^	27.62 ± 0.28^Ac^	27.69 ± 0.28^Ac^	27.65 ± 0.15^Ac^	27.65 ± 0.15^Ac^
	SCN12‐SH5	28.65 ± 0.04^Ac^	28.10 ± 0.04^Bc^	28.08 ± 0.04^Bc^	28.08 ± 0.06^Bc^	28.06 ± 0.20^Bc^
	SCN12‐SH14	28.88 ± 0.63^Abc^	28.81 ± 0.12^Ab^	28.76 ± 0.12^Ab^	28.73 ± 0.05^Ab^	28.69 ± 0.11^Ab^
	SCN9‐Mix	29.91 ± 0.72^Aab^	29.77 ± 0.15^Aa^	29.65 ± 0.23^Aa^	29.61 ± 0.12^Aa^	29.59 ± 0.32^Aa^
Turbidity	SCN9‐Control	0.638 ± 0.001^Bab^	0.653 ± 0.004^Bd^	0.694 ± 0.006^Aa^	0.701 ± 0.020^Ab^	0.711 ± 0.002^Ac^
	SCN12‐Control	0.645 ± 0.010^Cab^	0.676 ± 0.003^Bc^	0.764 ± 0.011^Aa^	0.779 ± 0.004^Aa^	0.777 ± 0.010^Aa^
	SCN9‐SH10	0.678 ± 0.042^Ca^	0.716 ± 0.011^BCa^	0.762 ± 0.004^Aa^	0.773 ± 0.005^Aa^	0.682 ± 0.005^Cd^
	SCN12‐SH5	0.643 ± 0.005^Eab^	0.683 ± 0.000^Dc^	0.714 ± 0.004^Cb^	0.730 ± 0.011^Bab^	0.754 ± 0.001^Ab^
	SCN12‐SH14	0.656 ± 0.004^Bab^	0.697 ± 0.000^ABb^	0.724 ± 0.025^Aab^	0.731 ± 0.042^Aab^	0.755 ± 0.000^Ab^
	SCN9‐Mix	0.614 ± 0.016^Db^	0.645 ± 0.001^Cd^	0.699 ± 0.010^Bb^	0.716 ± 0.016^ABb^	0.744 ± 0.014^Ab^

*Note:* Different lowercase letters indicate significant differences among sour cherry nectar samples, whereas different uppercase letters indicate significant differences across storage time (*p* < 0.05).

Abbreviations: SCN, Sour cherry nectar; SCN12‐SH14, SCN containing 12% sucrose inoculated with *Lactiplantibacillus pentosus* SH14; SCN12‐SH5, SCN containing 12% sucrose inoculated with *Lactiplantibacillus plantarum* SH5; SCN9‐Control and SCN12‐Control, Noninoculated SCN samples containing 9% and 12% sucrose, respectively; SCN9‐Mix, SCN containing 9% sucrose inoculated with a mixed culture of SH5, SH10, and SH14; SCN9‐SH10, SCN containing 9% sucrose inoculated with *Limosilactobacillus fermentum* SH10.

During storage, °Brix values remained nearly constant in all samples. Only SCN9–SH10 showed a slight reduction from 25.33 to 24.93 °Brix over 28 days, whereas the other samples exhibited negligible variation. This confirms that fermentation‐induced reductions in soluble solids occurred prior to storage and that subsequent changes during refrigerated storage were minimal.

Similar reductions in soluble solids due to fermentation have been reported in earlier research. In this context, Akman et al. (2025) reported total soluble solid content values for lactic acid‐fermented apricot juices ranging from 20.80 to 22.27 °Brix. Additionally, Güney and Güngörmüşler (2021) observed °Brix decreases of 0.7%–2.3% in a mixed vegetable juice (artichoke, pineapple, zucchini, spinach, cucumber) fermented with *L. paracasei* , *L. rhamnosus* , *L. plantarum* , and *B. animalis* subsp. lactis, while Zandi et al. (2016) found decreases of approximately 1%–1.5% in carrot–sugarcane–apple juice blends fermented with *L. casei* at different concentrations.

### 3.3 pH

Table 1 presents changes in the pH of lacto‐fermented sour cherry‐based beverages during cold storage. Throughout the 28‐day period, the pH of the nectar ranged between 3.54 and 3.62. The lowest pH values were consistently observed in the control samples, whereas the addition of SH10, SH5, SH14, or Mix cultures resulted in significantly higher pH levels than the controls (*p* < 0.05). The observed pH increase may be attributed to decarboxylation, as malic and malonic acids, the primary organic acids in sour cherry varieties (Sokół‐Łętowska), both contain two carboxyl groups. Comparable findings have been reported in the literature. For instance, Mousavi et al. (2011) observed that pomegranate juices fermented with *L. plantarum* and *L. delbrueckii* showed increased pH values and decreased total acidity, which was linked to the utilization of citric acid as a primary carbon source by the starter cultures. Likewise, Dimitrovski et al. (2015) demonstrated that apple juices adjusted to initial pH levels of 3.5, 4.2, and 5.1 and fermented with *L. plantarum* PCS 26 exhibited elevated pH after fermentation, reaching 7.0 and 4.7 in the samples initially set at 5.1 and 4.2, respectively. Although the reductions in pH observed in all sour cherry nectar samples during storage were relatively minor, they were statistically significant (*p* < 0.05). In contrast, no significant differences were observed in the control samples during the storage period.

### 3.4 Dry Matter

The variations in dry matter content of lacto‐fermented sour cherry‐based beverages during cold storage are summarized in Table 1. At the beginning of the study, the dry matter content of the samples ranged between 27.84% and 30.75%. Following fermentation, a decrease in dry matter was observed in all freshly prepared lactic acid fermented samples except SCN9‐Mix, with the most pronounced reductions detected in SCN9‐SH10 and SCN12‐SH5. During the storage period, all nectar samples exhibited gradual and statistically significant reductions in dry matter content compared to their initial values (*p* < 0.05). These reductions were strongly correlated with decreases in °Brix values. Similar findings have been documented in earlier studies. Resende Oliveira et al. (2018), for example, reported that alcoholic lacto‐fermented beverages derived from sugarcane showed decreases in dry matter content after fermentation. Conversely, Yeğin and Üren (2008), who investigated biogenic amine formation in traditional lacto‐fermented beverages (boza), found no association between fermentation and dry matter content. Likewise, Manga et al. (2019) demonstrated that raffia‐based beverages fermented with *Lactobacillus fermentum* and *Bifidobacterium bifidum* exhibited varying degrees of dry matter change depending on fermentation conditions. Similarly, Akman et al. (2025) reported a 6.95%–8.14% reduction in the dry matter content of apricot‐based lacto‐fermented beverages after 60 days of storage compared with freshly prepared samples.

### 3.5 Turbidity

Turbidity in liquids arises from suspended particles such as cells, proteins, or other insoluble materials. During fermentation, it indicates the presence of microbial cells, including yeast, as well as additional particulate matter (Ferreira et al. 2023). The variations in turbidity values of lactic acid fermented sour cherry‐based beverages during cold storage are presented in Table 1. Overall, turbidity values ranged between 0.614 and 0.777. Among the samples, SCN9–Mix exhibited the lowest turbidity throughout storage, except on days 14, 21, and 28, whereas SCN9–SH10 recorded the highest values (*p* < 0.05) on days 0 and 7. Conversely, SCN12–Control displayed the highest turbidity levels on days 14, 21, and 28. Significant increases (*p* < 0.05) in turbidity were also observed in SCN12–SH5, SCN12–SH14, and SCN9–Mix samples as storage progressed. Given that sour cherry juice is classified as a clarified fruit juice, turbidity is considered an undesirable quality attribute in such products. Consistent with the present results, Navruz et al. (2016) reported that sour cherry juice concentrates enriched with gallic acid and various plant extracts exhibited increased turbidity during storage at different temperatures. Likewise, Ertan et al. (2019) observed rising turbidity levels in sour cherry juices during storage. Huang et al. (2019) reported that the enzymatic activity of LAB on fruit cell walls alters the composition and molecular weight of polysaccharides, thereby influencing turbidity.

### 3.6 Color

The changes in color parameters and total color differences of lactic acid fermented sour cherry‐based beverages during cold storage are presented in Table 2. Statistically significant differences (*p* < 0.05) were observed in the color parameters of the samples following fermentation. The L* values of the samples ranged between 14.97 and 16.66, a* values between 12.80 and 18.21, b* values between −3.05 and −5.78, and ΔE* values between 1.69 and 17.66. At the beginning of storage, the highest L* and b* values were recorded in sample SCN12–SH5. However, no significant changes (*p* > 0.05) were observed in the color parameters of the samples during storage. The most remarkable change observed after fermentation was the substantial increase in a* values and the simultaneous shift of b* values toward less negative numbers, particularly in SCN9–SH10 and SCN12–SH5 samples. While control samples exhibited a* values around 13, fermented samples reached values above 18 (SCN9–SH10 and SCN12–SH5), indicating a pronounced enhancement in red coloration. Similarly, b* values shifted from approximately −5.7 in controls to around −3.1 in these samples, suggesting a transition from darker bluish‐red tones to brighter and more vivid red hues. Compared with the control nectars, all probiotic‐supplemented samples exhibited higher L* values, except for SCN12–SH5 at days 0 and 28. Similarly, a* and b* values increased after fermentation and remained higher than those of the controls throughout storage. This phenomenon is strongly associated with the degradation of anthocyanins and flavonoids at the end of the fermentation process (Rios‐Corripio and Guerrero‐Beltrán 2020). Moreover, ∆E* values indicated clear color differences between control and probiotic nectars, which were perceptible to the human eye (∆E* > 3) (Yavuz et al. 2022). Among the samples, SCN9–SH10 and SCN12–SH5 showed greater color differences relative to their control beverages, whereas SCN12–SH14 and SCN9–Mix exhibited smaller differences. Since the pH of all samples remained within a narrow acidic range (3.54–3.63), the pronounced differences observed in L*, a*, b*, and ΔE* values cannot be attributed to acidification effects. Samples SH10 and SH5 exhibited markedly higher a* and ΔE* values despite having lower TPC than the controls, while simultaneously presenting substantially higher DPPH and CUPRAC activities. This indicates that fermentation did not increase the total amount of phenolics but likely enhanced the availability and reactivity of phenolic compounds involved in color expression. In contrast, SH14 showed exceptionally high CUPRAC values but minimal color change, demonstrating that antioxidant capacity alone does not directly govern color modification. According to Degrain et al. (2020), the observed changes were likely due to pH fluctuations during fermentation, which led to the formation of new compounds. Comparable trends have been reported in other fruit‐based beverages. For instance, mulberry juice fermented with *Lactobacillus* strains demonstrated decreases in L* and b* values but an increase in a* values, which was attributed to elevated monomeric anthocyanin concentrations during fermentation (Kwaw, Ma, et al. 2018). Similarly, Pereira et al. (2011) observed that cashew apple juices adjusted to different initial pH levels and subsequently fermented with LAB cultures showed reductions in L* and a* values, whereas b* values increased after fermentation.

**TABLE 2:** Color attributes of lactic acid fermented sour cherry beverages during 28 days of storage, assessed at 7‐day intervals.

Parameters	Sample name	Day 0	Day 7	Day 14	Day 21	Day 28
L*	SCN9‐Control	15.24 ± 0.05^Ad^	15.15 ± 0.22^Ac^	15.18 ± 1.05^Aa^	14.97 ± 0.34^Ab^	15.08 ± 0.49^Ac^
	SCN12‐Control	15.56 ± 0.10^Acb^	15.20 ± 0.31^Ac^	15.24 ± 0.24^Aa^	15.12 ± 0.12^Ab^	15.78 ± 0.35^Abac^
	SCN9‐SH10	16.66 ± 0.15^Aa^	16.57 ± 0.65^Aa^	16.57 ± 0.35^Aa^	16.49 ± 0.37^Aa^	16.49 ± 0.08^Aa^
	SCN12‐SH5	15.43 ± 0.34^Ad^	15.41 ± 0.37^Ac^	15.45 ± 0.20^Aa^	15.45 ± 0.60^Aba^	15.47 ± 0.14^Abc^
	SCN12‐SH14	15.98 ± 0.12^Ab^	15.95 ± 0.13^Aba^	15.91 ± 0.21^Aa^	15.86 ± 0.20^Aba^	15.83 ± 0.10^Aba^
	SCN9‐Mix	15.24 ± 0.25^Ade^	15.23 ± 0.21^Ac^	15.25 ± 0.62^Aa^	15.31 ± 0.46^Ab^	15.30 ± 0.21^Abc^
a*	SCN9‐Control	13.00 ± 0.22^Ad^	12.94 ± 0.35^Ad^	13.02 ± 0.14^Ad^	13.00 ± 0.54^Ad^	13.00 ± 0.18^Ad^
	SCN12‐Control	12.86 ± 0.10^Ad^	12.80 ± 0.25^Ad^	12.95 ± 0.04^Ad^	12.86 ± 0.42^Ad^	12.89 ± 0.15^Ad^
	SCN9‐SH10	18.15 ± 0.24^Aa^	18.21 ± 0.03^Aa^	18.20 ± 0.04^Aa^	18.10 ± 0.22^Aa^	18.02 ± 0.15^Aa^
	SCN12‐SH5	18.20 ± 0.02^Aa^	18.20 ± 0.34^Aa^	18.15 ± 0.08^Aa^	18.06 ± 0.13^Aa^	18.11 ± 0.20^Aa^
	SCN12‐SH14	14.23 ± 0.31^Ac^	14.11 ± 0.18^Ac^	14.05 ± 0.12^Ac^	14.00 ± 0.20^Ac^	14.11 ± 0.22^Ac^
	SCN9‐Mix	15.62 ± 0.10^Ab^	15.63 ± 0.03^Ab^	15.59 ± 0.17^Ab^	15.60 ± 0.15^Ab^	15.49 ± 0.10^Ab^
b*	SCN9‐Control	−5.78 ± 0.10^Ac^	−5.67 ± 0.48^Ac^	−5.71 ± 0.41^Ac^	−5.74 ± 0.38^Ac^	−5.69 ± 0.15^Ac^
	SCN12‐Control	−5.65 ± 0.21^Ac^	−5.50 ± 0.11^Ac^	−5.65 ± 0.21^Ac^	−5.50 ± 0.35^Ac^	−5.60 ± 0.20^Ac^
	SCN9‐SH10	−3.24 ± 0.12^Aa^	−3.31 ± 0.28^Aa^	−3.29 ± 0.14^Aa^	−3.31 ± 0.12^Aa^	−3.28 ± 0.09^Aa^
	SCN12‐SH5	−3.05 ± 0.30^Aa^	−3.14 ± 0.11^Aa^	−3.09 ± 0.27^Aa^	−3.12 ± 0.10^Aa^	−3.10 ± 0.17^Aa^
	SCN12‐SH14	−4.33 ± 0.14^Ab^	−4.25 ± 0.25^Ab^	−4.21 ± 0.25^Ab^	−4.20 ± 0.20^Ab^	−4.22 ± 0.32^Ab^
	SCN9‐Mix	−4.20 ± 0.10^Ab^	−4.17 ± 0.34^Ab^	−4.18 ± 0.14^Ab^	−4.18 ± 0.34^Ab^	−4.19 ± 0.48^Ab^
ΔE*	SCN9‐SH10	17.50	17.66	17.31	17.11	16.51
	SCN12‐SH5	17.27	17.07	16.63	16.36	16.50
	SCN12‐SH14	1.89	1.91	1.87	1.77	1.69
	SCN9‐Mix	4.69	4.74	4.47	4.66	4.25

*Note:* Different lowercase letters in the tables indicate significant differences among sour cherry beverage formulations, whereas different uppercase letters indicate significant differences across storage time (two‐way ANOVA, Tukey's multiple comparison test, *p* < 0.05).

Abbreviations: SCN, Sour cherry nectar; SCN12‐SH14, SCN containing 12% sucrose inoculated with *Lactiplantibacillus pentosus* SH14; SCN12‐SH5, SCN containing 12% sucrose inoculated with *Lactiplantibacillus plantarum* SH5; SCN9‐Control and SCN12‐Control, Noninoculated SCN samples containing 9% and 12% sucrose, respectively; SCN9‐Mix, SCN containing 9% sucrose inoculated with a mixed culture of SH5, SH10, and SH14; SCN9‐SH10, SCN containing 9% sucrose inoculated with *Limosilactobacillus fermentum* SH10.

### 3.7 Bioactive Properties and Antioxidant Activity

The changes in the bioactive properties of lactic acid fermented sour cherry‐based beverages during cold storage are presented in Table 3. At the beginning of storage, the total phenolic content of the samples ranged from 214.75 to 353.67 mg GAE/100 mL. The TPC of the lactic acid fermented samples was lower than that of the control group, indicating the degradation of phenolic compounds during fermentation. Moreover, a time‐dependent decline in TPC was observed in all samples throughout storage. The decline in TPC observed in fermented fruits may result from the decarboxylation of specific phenolic compounds (Méndez‐Galarraga et al. 2025; Zeng et al. 2021). It has been confirmed that complex phenolic compounds can be hydrolyzed into simpler forms by microbial hydrolytic enzymes, although the capacity to transform phenols varies among bacterial genera (Peng et al. 2024).

**TABLE 3:** TPC, DPPH scavenging, and CUPRAC activities of lactic acid fermented sour cherry beverages during 28 days of storage, assessed at 7‐day intervals.

Name of analysis	Sample name	Day 0	Day 7	Day 14	Day 21	Day 28
TPC (mg GAE/100 mL)	SCN9‐Control	353.48 ± 5.42^Aa^	312.58 ± 3.52^Ba^	305.90 ± 1.73^Ba^	286.37 ± 6.96^Ca^	266.00 ± 2.93^Da^
	SCN12‐Control	353.67 ± 4.24^Aa^	315.71 ± 3.66^Ba^	302.50 ± 5.98^BCa^	291.54 ± 3.99^Ca^	264.06 ± 7.21^Da^
	SCN9‐SH10	295.72 ± 7.26^Abc^	274.35 ± 2.00^Bb^	253.31 ± 2.79^Cb^	232.61 ± 1.04^Dbc^	224.10 ± 2.02^Db^
	SCN12‐SH5	288.37 ± 3.98^Ac^	259.66 ± 2.52^Bc^	236.95 ± 1.90^Cc^	222.43 ± 3.02^Dc^	214.75 ± 3.28^Db^
	SCN12‐SH14	301.56 ± 2.26^Ab^	275.68 ± 0.29^Bb^	256.15 ± 2.02^Cb^	238.62 ± 4.65^Db^	221.43 ± 3.33^Eb^
	SCN9‐Mix	293.255 ± 3.48^Abc^	277.81 ± 4.21^Bb^	261.35 ± 1.44^Cb^	229.24 ± 6.63^Dbc^	217.31 ± 2.98^Eb^
DPPH scavenging activity (mg TE/100 mL)	SCN9‐Control	252.71 ± 0.72^Ad^	251.36 ± 0.14^Ad^	248.22 ± 0.11^ABd^	244.59 ± 3.97^Bc^	223.46 ± 0.32^Cd^
	SCN12‐Control	251.64 ± 0.58^Ad^	251.78 ± 0.26^Ac^	249.35 ± 0.23^Bc^	242.84 ± 1.46^Cc^	229.55 ± 0.78^Dcd^
	SCN9‐SH10	273.69 ± 0.52^Aa^	270.80 ± 0.07^Aa^	269.51 ± 0.14^Aa^	267.65 ± 0.11^Aa^	240.24 ± 7.18^Bb^
	SCN12‐SH5	263.98 ± 0.04^Ab^	260.80 ± 0.07^ABb^	259.32 ± 0.04^ABb^	257.95 ± 0.04^Bb^	238.99 ± 4.25^Cbc^
	SCN12‐SH14	274.02 ± 0.11^Aa^	270.90 ± 0.11^Ba^	269.40 ± 0.11^Ca^	267.63 ± 0.18^Da^	250.02 ± 0.62^Ea^
	SCN9‐Mix	257.53 ± 1.08^Ac^	245.77 ± 0.14^Be^	231.60 ± 0.27^Ce^	229.86 ± 1.65^Cd^	213.54 ± 0.77^De^
CUPRAC (mg TE/100 mL)	SCN9‐Control	994.47 ± 9.26^Ad^	982.80 ± 10.10^Aa^	962.97 ± 33.76^ABd^	946.63 ± 11.25^ABc^	924.46 ± 12.62^Bc^
	SCN12‐Control	996.31 ± 4.42^Ad^	985.70 ± 7.90^ABc^	959.56 ± 12.45^BCd^	947.77 ± 5.60^CDc^	931.00 ± 15.25^Dc^
	SCN9‐SH10	1232.50 ± 33.39^Ab^	1216.16 ± 52.66^Aa^	1172.99 ± 3.50^ABb^	1132.15 ± 16.54^Bb^	1109.98 ± 12.13^Bb^
	SCN12‐SH5	1108.82 ± 12.29^Ac^	1090.15 ± 4.04^Ab^	1084.31 ± 38.56^Ac^	1081.98 ± 37.04^Ab^	1069.14 ± 17.62^Ab^
	SCN12‐SH14	1576.71 ± 68.71^Aa^	1262.83 ± 35.93^Ba^	1258.17 ± 35.06^Ba^	1257.00 ± 24.50^Ba^	1243.48 ± 18.19^Ba^
	SCN9‐Mix	1078.42 ± 34.81^Acd^	1053.09 ± 27.45^ABbc^	998.63 ± 15.71^BCd^	965.05 ± 36.17^Cc^	936.34 ± 26.11^Cc^

*Note:* Different lowercase letters in the tables indicate significant differences among sour cherry beverage formulations, whereas different uppercase letters indicate significant differences across storage time (two‐way ANOVA, Tukey's multiple comparison test, *p* < 0.05).

Abbreviations: SCN, Sour cherry nectar; SCN12‐SH14, SCN containing 12% sucrose inoculated with *Lactiplantibacillus pentosus* SH14; SCN12‐SH5, SCN containing 12% sucrose inoculated with *Lactiplantibacillus plantarum* SH5; SCN9‐Control and SCN12‐Control, Noninoculated SCN samples containing 9% and 12% sucrose, respectively; SCN9‐Mix, SCN containing 9% sucrose inoculated with a mixed culture of SH5, SH10, and SH14; SCN9‐SH10, SCN containing 9% sucrose inoculated with *Limosilactobacillus fermentum* SH10.

The DPPH radical scavenging activity of the nectars ranged between 213.54 and 274.02 mg TE/100 mL, while CUPRAC values were between 924.46 and 1576.71 mg TE/100 mL (Table 3). Based on these results, the antioxidant capacity of the lactic acid fermented samples was higher than that of the control. This trend was consistent for all samples in the CUPRAC assay and, with the exception of SCN9–Mix at days 7, 14, 21, and 28, also in the DPPH assay. These findings suggest that fermentation enhanced the antioxidant capacity of the beverages, and notably, the values of the lactic acid fermented samples remained consistently higher than those of the control throughout storage. These findings suggest that lactic acid bacteria fermentation enhanced the DPPH radical scavenging activity of sour cherry–based beverages, consistent with results reported for fermented strawberry juice, likely due to the release of more soluble phenolic compounds (Chen et al. 2023).

Although TPC decreased in all samples during storage, the magnitude of this decrease and its impact on antioxidant capacity were clearly strain‐dependent. Among the fermented samples, SCN12‐SH14 exhibited the highest DPPH and CUPRAC values throughout storage despite a continuous reduction in TPC, followed by SCN9‐SH10 and SCN12‐SH5. This indicates that SH14 and SH10 strains were more effective in transforming complex phenolic compounds into smaller molecules with higher reducing and radical scavenging capacity. In contrast, the SCN9‐Mix sample showed the greatest decline in both TPC and antioxidant activity over time, suggesting possible inter‐strain competition that limited efficient phenolic biotransformation. While the control samples showed a parallel decrease in TPC, DPPH, and CUPRAC values, fermented samples maintained significantly higher antioxidant activities than controls at all storage times.

The antioxidant and antiradical activities of fermented fruit‐based beverages have been shown to vary depending on fermentation conditions, primarily due to changes in phytochemical composition. Probiotic activity has been suggested to promote the depolymerization of macromolecular phenolics or their conversion into smaller, more bioactive compounds (Wu et al. 2020). For instance, Sina et al. (2021) reported that fermented *Morinda citrifolia* juice exhibited stronger DPPH scavenging activity than fresh juice. Similarly, Laosee et al. (2022) demonstrated that winter melon and pineapple juices fermented with combinations of *L. plantarum* , *L. salivarius* , and *Saccharomyces boulardii* exhibited variable antioxidant capacities depending on the starter cultures applied. The bioactivity of phenolic compounds is strongly influenced by the number and position of hydroxyl groups, which determine their ability to act as reducing agents, radical scavengers, and singlet oxygen quenchers (de Souza et al. 2019; Verón et al. 2019). Enhancement of antioxidant activity through LAB fermentation has been attributed to improved functional properties of polyphenols with proton‐donating capacity (dos Santos Filho et al. 2019). In line with these observations, Wu et al. (2020) reported that apple juice fermented with different LAB strains exhibited decreased total phenolic content but increased antioxidant capacity following fermentation. Méndez‐Galarraga et al. (2025) reported that fermentation‐induced changes in phenolic profiles are largely driven by strain‐specific enzymes that modify phenolics through reactions such as deglycosylation, demethylation, dehydroxylation, ester cleavage, reduction, isomerization, ring fission, and decarboxylation. These enzymatic activities may also release bound phenolics by degrading cell wall components or by depolymerizing complex structures such as tannins. The antioxidant activity of phenolic compounds is strongly related to their chemical structure rather than their total quantity. Flavonoid‐type phenolics generally exhibit higher antioxidant capacity than nonflavonoids, while glycosylated and conjugated forms display lower activity compared to their free counterparts (Paventi et al. 2025). This mechanism explains the present findings where TPC values decreased during storage, yet DPPH and CUPRAC activities remained high, particularly in SH10, SH5, and SH14 samples. Such behavior suggests that fermentation promoted the conversion of conjugated phenolics into more reactive free forms, thereby enhancing antioxidant efficiency without increasing total phenolic content.

### 3.8 Phenolic Profile

**FIGURE 2:**
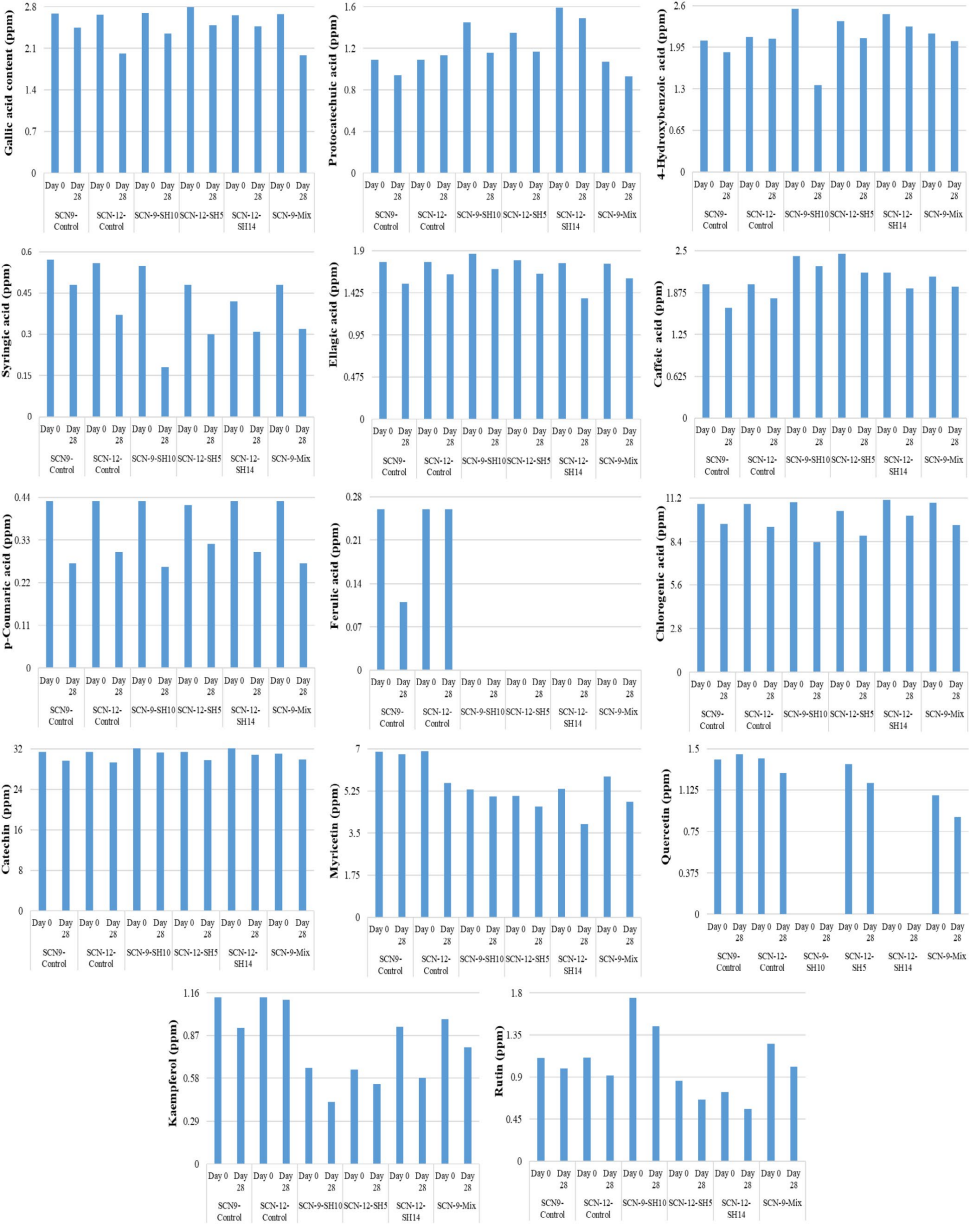
Phenolic and flavonoid acid content of lactic acid fermented sour cherry beverages, analyzed on Day 0 (freshly prepared) and after 28 days of storage throughout the gastrointestinal digestion phases. SCN, Sour cherry nectar; SCN12‐SH14, SCN containing 12% sucrose inoculated with *Lactiplantibacillus pentosus* SH14; SCN12‐SH5, SCN containing 12% sucrose inoculated with *Lactiplantibacillus plantarum* SH5; SCN9‐Control and SCN12‐Control, Noninoculated SCN samples containing 9% and 12% sucrose, respectively; SCN9‐Mix, SCN containing 9% sucrose inoculated with a mixed culture of SH5, SH10, and SH14; SCN9‐SH10, SCN containing 9% sucrose inoculated with *Limosilactobacillus fermentum* SH10.

Changes in the phenolic profiles of lactic acid fermented sour cherry‐based beverages during cold storage are presented in Figure 2. The identified phenolic acids included gallic acid (Day 0: 2.65–2.69 ppm; Day 28: 1.98–2.49 ppm), protocatechuic acid (Day 0: 1.07–1.59 ppm; Day 28: 0.93–1.49 ppm), 4‐hydroxybenzoic acid (Day 0: 2.05–2.55 ppm; Day 28: 1.36–2.27 ppm), syringic acid (Day 0: 0.42–0.57 ppm; Day 28: 0.18–0.48 ppm), ellagic acid (Day 0: 1.75–1.86 ppm; Day 28: 1.36–1.69 ppm), caffeic acid (Day 0: 2.00–2.45 ppm; Day 28: 1.65–2.27 ppm), p‐coumaric acid (Day 0: 0.42–0.43 ppm; Day 28: 0.26–0.32 ppm), ferulic acid (Day 0: 0.00–0.26 ppm; Day 28: 0.00–0.26 ppm), and chlorogenic acid (Day 0: 10.36–11.05 ppm; Day 28: 8.35–10.06 ppm). The detected flavonoids were catechin (Day 0: 31.05–32.90 ppm; Day 28: 29.33–31.20 ppm), myricetin (Day 0: 5.05–6.89 ppm; Day 28: 3.88–6.77 ppm), quercetin (Day 0: 0.00–1.41 ppm; Day 28: 0.00–1.45 ppm), kaempferol (Day 0: 0.64–1.13 ppm; Day 28: 0.42–1.11 ppm), and rutin (Day 0: 0.74–1.75 ppm; Day 28: 0.56–1.44 ppm). Significant differences in phenolic composition were observed between the lactic acid fermented samples and the control group after fermentation. Nevertheless, a gradual decrease in the overall phenolic profile was noted in all samples by Day 28 compared with Day 0. Catechin was identified as the predominant compound across all samples, followed by chlorogenic acid and myricetin.

Overall, variations in phenolic acid content were observed among freshly prepared fruit juices, depending on the sugar added during fermentation and the type of microorganism used. Although no consistent trend was identified, compared to the control samples, phenolic acid levels on Day 0 either decreased, increased, or remained unchanged. For instance, gallic and ellagic acid contents increased in SCN9‐SH10 and SCN12‐SH5, whereas they decreased in SCN12‐SH14 and SCN9‐Mix. Regarding protocatechuic acid, all samples, except SCN9‐Mix, showed higher levels than their controls on Day 0. Similarly, 4‐hydroxybenzoic and caffeic acids increased in all fermented juices, while syringic and ferulic acids decreased. p‐Coumaric acid remained stable in most samples except SCN12‐SH5, where it decreased, whereas chlorogenic acid increased in all juices except SCN12‐SH5. Regarding flavonoids, catechin content remained unchanged in SCN12‐SH5 but increased in SCN9‐SH10 and SCN12‐SH14 on Day 0, while myricetin, quercetin, and kaempferol contents decreased across all fermented juices. Rutin levels increased in SCN9‐SH10 and SCN9‐Mix, but decreased in SCN12‐SH5 and SCN12‐SH14.

After 28 days of storage, gallic acid decreased in SCN9‐SH10 and 4‐hydroxybenzoic acid samples but increased in the other juices. Protocatechuic acid increased in SCN9‐SH10, SCN12‐SH5, and SCN12‐SH14, whereas a reduction was noted in SCN9‐Mix. Syringic acid decreased after fermentation, and ferulic acid was not detected in any fermented samples. Ellagic acid decreased in SCN12‐SH14 but increased in the other samples. Caffeic acid increased in all fermented juices compared to controls, while p‐coumaric acid decreased in SCN9‐SH10 and SCN12‐SH5 but remained unchanged in the others. Chlorogenic acid levels decreased in SCN9‐SH10 and SCN12‐SH5, whereas increases were observed in the other two juices. Regarding flavonoids, myricetin, quercetin, and kaempferol contents decreased in all fermented juices after 28 days of storage. In contrast, catechin and rutin levels increased in SCN9‐SH10 and SCN9‐Mix, while decreases were recorded in SCN12‐SH5 and SCN12‐SH14.

After 28 days of storage, gallic acid levels decreased in SCN9‐SH10 and SCN9‐Mix, whereas 4‐hydroxybenzoic acid declined only in SCN9‐SH10 but increased in all other samples. Protocatechuic acid content increased in SCN9‐SH10, SCN12‐SH5, and SCN12‐SH14, while a reduction was observed in SCN9‐Mix. Syringic acid decreased after fermentation, and ferulic acid was not detected in any fermented samples. Ellagic acid content decreased in SCN12‐SH14 but increased in the remaining samples. Caffeic acid levels rose in all lactic acid–fermented juices compared to their controls. In contrast, p‐coumaric acid decreased in SCN9‐SH10 and SCN12‐SH5 but remained unchanged in the others. Chlorogenic acid levels declined in SCN9‐SH10 and SCN12‐SH5, while increases were noted in the other two fermented juices. Regarding flavonoids, myricetin, quercetin, and kaempferol contents decreased in all fermented samples after 28 days of storage. Conversely, catechin and rutin levels increased in SCN9‐SH10 and SCN9‐Mix, whereas reductions were recorded in SCN12‐SH5 and SCN12‐SH14.

The phenolic composition of fruit juices is influenced not only by intrinsic factors such as fruit type, ripeness, and climatic or geographical conditions, but also by processing‐related parameters, including thermal treatment, fermentation, storage, and the use of additives (Burin et al. 2010). For example, in a study on hardaliye (a traditional fermented beverage), samples stored for 60 days at 0°C, 4°C, or 20°C exhibited increases in gallic acid, resveratrol, abscisic acid, and trans‐p‐coumaric acid depending on storage temperature (Aşkin and Atik 2016). Similarly, Li et al. (2021) reported that 48 h of fermentation of blueberry juice with different LAB strains resulted in increases of 136%, 71%, and 38% in rutin, myricetin, and gallic acid contents, respectively, while p‐hydroxybenzoic acid and caffeic acid levels decreased. In another study, Hu et al. (2019) demonstrated that fermentation of carrot juice with *L. rhamnosus* GG enhanced the levels of free quercetin, gallic acid, catechinic acid, chlorogenic acid, and kaempferol.

### 3.9 Total Caloric Value

Changes in the caloric values of lactic acid fermented sour cherry‐based beverages during cold storage are presented in Table 4. At the beginning of storage, caloric values ranged from 32.00 to 72.00 kcal/100 mL, with statistically significant differences among the samples (*p* < 0.05). Following fermentation, significant reductions (*p* < 0.05) in caloric values were observed in all lactic acid fermented samples compared to the control group. Among them, SCN9–SH10 exhibited the lowest caloric value, which was attributed to its lower initial sugar content combined with higher starter culture activity.

**TABLE 4:** Changes in caloric value, sugar content, LAB viability, and antimicrobial activity against *Bacillus cereus* in lactic acid fermented sour cherry beverages over 28 days of storage at 7‐day intervals.

Analysis name	Sample name	Day 0	Day 7	Day 14	Day 21	Day 28
Calorie value (kcal/100 mL)	SCN9‐Control	59.50 ± 0.45^Ab^	59.50 ± 0.45^Ab^	59.45 ± 0.40^Ab^	59.40 ± 0.40^Ab^	59.40 ± 0.27^Ab^
	SCN12‐Control	72.00 ± 0.64^Aa^	72.00 ± 0.65^Aa^	72.02 ± 0.45^Aa^	72.02 ± 0.33^Aa^	72.03 ± 0.32^Aa^
	SCN9‐SH10	32.00 ± 0.67^Ae^	31.98 ± 0.68^Ae^	31.90 ± 0.58^Ae^	31.85 ± 0.58^Af^	31.80 ± 0.73^Af^
	SCN12‐SH5	44.20 ± 0.50^Ac^	44.15 ± 0.48^Ac^	44.08 ± 0.45^Ac^	43.93 ± 0.50^Ad^	43.90 ± 0.55^Ad^
	SCN12‐SH14	40.50 ± 0.65^Ad^	40.43 ± 0.58^Ad^	40.33 ± 0.61^Ad^	40.20 ± 0.56^Ae^	40.10 ± 0.34^Ae^
	SCN9‐Mix	45.46 ± 0.41^Ac^	45.38 ± 0.38^Ac^	45.35 ± 0.37^Ac^	45.32 ± 0.37^Ac^	45.32 ± 0.36^Ac^
Fructose (g/100 mL)	SCN9‐Control	1.64 ± 0.07^Aa^	1.64 ± 0.07^Aa^	1.65 ± 0.10^Aa^	1.65 ± 0.11^Aa^	1.65 ± 0.11^Aa^
	SCN12‐Control	1.65 ± 0.05^Aa^	1.65 ± 0.05^Aa^	1.65 ± 0.05^Aa^	1.65 ± 0.05^Aa^	1.65 ± 0.05^Aa^
	SCN9‐SH10	1.09 ± 0.04^Ab^	1.09 ± 0.05^Ab^	1.09 ± 0.05^Ab^	1.09 ± 0.05^Ab^	1.09 ± 0.03^Ab^
	SCN12‐SH5	1.11 ± 0.03^Ab^	1.11 ± 0.03^Ab^	1.11 ± 0.03^Ab^	1.11 ± 0.03^Ab^	1.11 ± 0.02^Ab^
	SCN12‐SH14	1.11 ± 0.01^Ab^	1.11 ± 0.01^Ab^	1.12 ± 0.01^Ab^	1.11 ± 0.01^Ab^	1.11 ± 0.01^Ab^
	SCN9‐Mix	1.14 ± 0.06^Ab^	1.14 ± 0.05^Ab^	1.14 ± 0.06^Ab^	1.14 ± 0.05^Ab^	1.14 ± 0.05^Ab^
Sucrose (g/100 mL)	SCN9‐Control	10.31 ± 0.04^Ab^	10.31 ± 0.05^Ab^	10.30 ± 0.05^Ab^	10.31 ± 0.03^Ab^	10.31 ± 0.04^Ab^
	SCN12‐Control	13.17 ± 0.04^Aa^	13.16 ± 0.02^Aa^	13.17 ± 0.02^Aa^	13.17 ± 0.02^Aa^	13.17 ± 0.08^Aa^
	SCN9‐SH10	5.14 ± 0.26^Ae^	5.14 ± 0.28^Ae^	5.14 ± 0.18^Ae^	5.13 ± 0.19^Ae^	5.12 ± 0.18^Ae^
	SCN12‐SH5	8.08 ± 0.20^Ad^	8.05 ± 0.18^Ad^	8.00 ± 0.15^Ad^	7.94 ± 0.14^Ad^	7.94 ± 0.08^Ad^
	SCN12‐SH14	7.67 ± 0.20^Ad^	7.66 ± 0.22^Ad^	7.65 ± 0.22^Ad^	7.64 ± 0.21^Ad^	7.63 ± 0.21^Ad^
	SCN9‐Mix	8.86 ± 0.16^Ac^	8.84 ± 0.16^Ac^	8.84 ± 0.19^Ac^	8.83 ± 0.16^Ac^	8.82 ± 0.16^Ac^
LAB count (log CFU/mL)	SCN9‐SH10	9.94 ± 0.14^Aa^	9.32 ± 0.13^Ba^	8.44 ± 0.14^Cab^	7.43 ± 0.07^Db^	6.27 ± 0.06^Eb^
	SCN12‐SH5	9.85 ± 0.18^Aa^	9.35 ± 0.40^Aa^	8.32 ± 0.12^Bab^	7.36 ± 0.05^Cb^	5.99 ± 0.03^Dc^
	SCN12‐SH14	9.97 ± 0.07^Aa^	9.42 ± 0.34^Ba^	8.47 ± 0.11^Ca^	7.65 ± 0.02^Da^	6.46 ± 0.04^Ea^
	SCN9‐Mix	9.82 ± 0.11^Aa^	9.21 ± 0.45^Aa^	8.04 ± 0.26^Bb^	7.01 ± 0.10^Cc^	5.87 ± 0.09^Dc^
Inhition zone against *B. cereus* (mm)	Control	12.00 ± 0.00	—	—	—	—
	Control‐pH 5	7.50 ± 0.58	—	—	—	—
	SCN9‐Mix	11.50 ± 0.58	—	9.00 ± 0.00	8.00 ± 1.15	—

*Note:* Different lowercase letters in the tables indicate significant differences among sour cherry beverage formulations, whereas different uppercase letters indicate significant differences across storage time (two‐way ANOVA, Tukey's multiple comparison test, *p* < 0.05).

Abbreviations: SCN, Sour cherry nectar; SCN12‐SH14, SCN containing 12% sucrose inoculated with *Lactiplantibacillus pentosus* SH14; SCN12‐SH5, SCN containing 12% sucrose inoculated with *Lactiplantibacillus plantarum* SH5; SCN9‐Control and SCN12‐Control, Noninoculated SCN samples containing 9% and 12% sucrose, respectively; SCN9‐Mix, SCN containing 9% sucrose inoculated with a mixed culture of SH5, SH10, and SH14; SCN9‐SH10, SCN containing 9% sucrose inoculated with *Limosilactobacillus fermentum* SH10.

During storage, no significant changes (*p* > 0.05) in caloric values were detected in any sample, indicating limited bacterial activity under cold storage conditions. However, when compared with their respective controls, the reductions remained substantial throughout the storage period. For instance, after 28 days of storage, SCN9–SH10 and SCN12–SH14 showed decreases in caloric content of 46.22%–46.46% and 43.75%–44.32%, respectively. These results confirm that the major reduction in caloric content occurred during fermentation rather than storage. During food fermentation, microbial metabolism decreases plant‐derived monosaccharides and disaccharides, such as fructose, glucose, and sucrose, through catabolic pathways. This reduction in specific sugars may contribute to a lower glycemic index and improved tolerability of the product (Borrego‐Ruiz et al. 2024).

Since clarified fruit juices such as sour cherry juice contain negligible amounts of dietary fiber, fat, and protein, starter cultures primarily metabolize sugars as their main carbon source during fermentation, leading to marked reductions in caloric content. In line with this, Xu et al. (2020) reported that carrot juice fermented with *L. gasseri* isolates exhibited a decrease in caloric value due to microbial sugar utilization. Similarly, Hu et al. (2019) found that diabetic rats fed with carrot juice fermented by *L. rhamnosus* GG showed nearly 50% lower blood glucose levels compared to the control group, an effect attributed to reduced simple sugar and caloric content. Moreover, Johnson et al. (2016) demonstrated that dietary supplementation with lactic acid fermented blueberry juice alleviated obesity and hyperglycemia in mice, further highlighting the potential metabolic benefits of reduced‐calorie lactic acid fermented beverages.

### 3.10 Fructose and Sucrose Content

The changes in fructose and sucrose contents of lacto‐fermented sour cherry‐based beverages during cold storage are presented in Table 4. In both the lacto‐fermented samples and the control group, sucrose content was higher than fructose. Compared to the controls, sucrose levels decreased by approximately 34% in SCN9‐SH10 and by about 42% in SCN12‐SH14 during storage. Following fermentation, significant reductions (*p* < 0.05) were observed in both fructose and sucrose levels, with a more pronounced decrease in sucrose. Specifically, fructose contents decreased by nearly 34% in SCN9‐SH10 and by about 33% in SCN12‐SH5 and SCN12‐SH14 compared to their respective controls. Among the lacto‐fermented samples, SCN9‐SH10 exhibited the lowest concentrations of both fructose and sucrose. However, during storage, no significant changes (*p* > 0.05) were detected in the sugar contents of the samples. The reduction in sugar content of the lacto‐fermented beverages can be attributed to the utilization of sugars as carbon sources by the starter cultures during fermentation and their subsequent metabolic activities (Akman et al. 2025). Consistent with these findings, Xu et al. (2020) reported that carrot juices fermented with two different strains of *L. gasseri* showed up to 27% reductions in sugar levels, with sucrose, glucose, and fructose being metabolized in descending order of preference. Similar results were also reported by Hu et al. (2019). Moreover, the results indicated that all three LAB were able to metabolize soluble sugars in tomato juice, generating compounds such as acetic acid, lactic acid, and ethanol through glycolysis and the pentose phosphate pathway (Jiang et al. 2025). While the different strains generally consumed similar amounts of fructose, the main differences were observed in sucrose utilization, with SH10 consuming the most, followed by SH14, SH5, and Mix. Güney and Güngörmüşler (2021) reported that since sugars are not fully depleted by the end of fermentation, their residual presence during storage provides a substrate that supports slow bacterial growth and continued fermentation.

### 3.11 Total LAB Count

LAB counts are critical for fermentation quality and stability, with viable cell numbers serving as a reliable indicator of strain adaptability (Zhao et al. 2025). These variations in total LAB counts of lacto‐fermented sour cherry‐based beverages during cold storage are presented in Table 4. Following fermentation, LAB counts in the lacto‐fermented samples ranged from 9.82 to 9.97 log CFU/mL, with no statistically significant differences among the samples (*p* > 0.05). However, a gradual decline in LAB counts was observed in all samples throughout storage.

A gradual decline in LAB counts was observed in all samples during storage; however, strain‐dependent differences were evident at the end of the storage period. On Day 28, SCN9‐SH10 and SCN12‐SH14 maintained LAB counts of 6.27 and 6.46 log CFU/mL, respectively, remaining above the 10^6^ CFU/mL threshold required for probiotic potential. In contrast, SCN12‐SH5 and SCN9‐Mix decreased to 5.99 and 5.87 log CFU/mL, respectively, falling below this threshold. These results indicate that SH10 and particularly SH14 strains exhibited greater adaptability and survival under cold storage conditions compared to SH5 and the mixed culture. The lower survival observed in the mixed culture may be due to inter‐strain competition that affects long‐term viability. This strain‐dependent difference in survival can be attributed to the fact that the viability of probiotic microorganisms is strongly influenced by the environmental conditions of the final product, the characteristics of the food matrix, and the intrinsic properties of the strains, which must be maintained throughout storage (Matouskova et al. 2021). Moreover, Ricci et al. (2019) reported that cherry juice presents a challenging environment for microbial growth due to its low pH, high sugar and phenolic content, and the presence of malic acid.

Despite this decrease, the final LAB counts remained above 10^6^ CFU/mL, which is considered the threshold level for probiotic potential (Bagdat, Akman, et al. 2024; Bagdat, Kutlu, and Tornuk 2024; Süren et al. 2024). In a related study, natural sour cherry juice and sour cherry juice supplemented with yeast extract were fermented for 24 h using various Lactobacillus strains, after which LAB counts were evaluated. A significant reduction (*p* < 0.05) in LAB counts was reported in the natural sour cherry juice following fermentation, whereas the yeast extract‐supplemented compared to 7 log CFU/mL in the unsupplemented juice (Perjéssy et al. 2020).

### 3.12 Antimicrobial Activity

The antimicrobial activity of lacto‐fermented sour cherry‐based beverages during cold storage is presented in Table 4. In our study, the test microorganisms included *Staphylococcus aureus* , *Escherichia coli* O157:H7, *Bacillus cereus* , *Listeria monocytogenes* , *Candida albicans* , and *Saccharomyces cerevisiae* . The pH values of the lactofermented sour cherry‐based beverages were adjusted to 5, 6, and 7. Among the tested samples, only SCN9‐Mix (Day 0, Day 14, and Day 21) and the control samples (Control Day 0 and Control‐pH‐5 Day 0) exhibited antibacterial activity against *Bacillus cereus* , whereas no inhibitory effects were observed in the other samples. During storage, the inhibitory effect rapidly diminished and completely disappeared by Day 28. The decline and eventual disappearance of inhibitory activity during storage can be explained by the instability and progressive degradation of fermentation‐derived antimicrobial metabolites such as organic acids, hydrogen peroxide, and bacteriocin‐like peptides, coupled with the absence of ongoing metabolite synthesis after fermentation (Prabhurajeshwar and Chandrakanth 2017; Wang et al. 2025). The selective inhibition observed in SCN9‐Mix suggests a synergistic effect of metabolites produced by the mixed LAB culture. The coexistence of facultative heterofermentative species ( *L. plantarum* and *L. pentosus* ) with the obligate heterofermentative *L. fermentum* likely broadened the spectrum of antimicrobial metabolites produced, contributing to the enhanced inhibitory effect observed in mixed cultures. The simultaneous production of organic acids, hydrogen peroxide, and bacteriocin‐like peptides by mixed cultures likely intensified membrane disruption, oxidative stress, and intracellular pH imbalance in *B. cereus* , explaining the observed antimicrobial efficacy (Serna‐Cock et al. 2019). This may explain why antimicrobial activity was not detected in samples fermented with individual strains. The disappearance of antimicrobial activity of SCN‐9‐Mix during storage indicates that these metabolites are unstable under prolonged cold conditions and may degrade or lose activity over time, even though viable LAB counts and antioxidant capacity were still present. Furthermore, according to Wang et al. (2025), this decline in antimicrobial activity can be mechanistically explained by the instability of fermentation‐derived antimicrobial metabolites within the food matrix. Proteinaceous bacteriocin‐like peptides and other antimicrobial compounds are known to be highly sensitive to environmental factors such as pH changes, oxygen exposure, interactions with food components, and storage conditions. These factors may lead to denaturation, inactivation, or degradation of such metabolites over time, thereby reducing the observed antimicrobial efficacy.

Previous studies have highlighted that the antimicrobial properties of fruit‐based beverages can differ considerably. For example, Sina et al. (2021) reported that both fresh and fermented *Morinda citrifolia* juices displayed variable antibacterial effects against *S. aureus, Pseudomonas aeruginosa, Proteus mirabilis, Staphylococcus epidermidis, Proteus vulgaris, Streptococcus oralis, Enterococcus faecalis*, and *E. coli* . Similarly, extracts derived from blueberry juices fermented with *Bacillus amyloliquefaciens, L. brevis*, and *Starmerella bombicola* were shown to exert strong antimicrobial activity against skin pathogens such as *Brevibacterium linens, Propionibacterium acnes, B. cereus*, and *S. epidermidis* . Moreover, these findings are consistent with previous observations reported by Ankolekar et al. (2011), who investigated the inhibitory potential of fermented cherry extracts against *Helicobacter pylori* . In their study, only the fermented cherry extracts exhibited inhibitory activity, which was dependent on both sample volume and pH, highlighting the critical role of fermentation‐derived metabolites and acidity in modulating antimicrobial effects.

### 3.13 Cytotoxic Activity

The cytotoxic activity of lacto‐fermented sour cherry‐based beverages against the MCF‐7 cell line during refrigerated storage is presented in Table 5. In general, the beverages exhibited relatively low cytotoxic effects toward MCF‐7 breast cancer cells at the tested concentrations. At the onset of storage, freshly prepared nectar at the highest tested concentration (100 μL/mL) demonstrated the most pronounced cytotoxic activity in SCN12‐SH14 (*p* < 0.05), reducing cell viability to 87.64%. This was followed by SCN9‐SH10 (89.87%), SCN12‐SH5 (93.31%), and SCN9‐Mix (94.45%). At this concentration, lacto‐fermented samples exhibited significantly higher (*p* < 0.05) anticancer potential compared to their nonfermented counterparts. Nevertheless, by the end of storage (Day 28), the cytotoxic effect diminished, as reflected by increased cell viability values compared to those observed on Day 0, with the decline being more pronounced in the lacto‐fermented samples. These results are consistent with previous findings. Ravanbakhshian and Behbahani (2018) reported that garlic extract fermented with *Lactiplantibacillus plantarum* exhibited cytotoxic effects, underscoring its anticancer potential. Similarly, Vaithilingam et al. (2016) demonstrated that beetroot juice fermented for 48 h with a mixed culture of *Lactobacillus acidophilus* and *L. plantarum* reduced the viability of human lung cancer cells by 64%. Moreover, Rajendran et al. (2025) demonstrated that fermented pomegranate juice with *Lactobacillus pentosus* and *Lactobacillus fermentum* interferes with the normal cell cycle progression of HT‐29 cells, which may underlie its potential therapeutic role in cancer treatment. Furthermore, Mostafa et al. (2020) reported that probiotic date juice containing *Lactobacillus acidophilus* and *L. sakei* exhibited in vitro antitumor activity against human larynx carcinoma (Hep‐2) cells, while showing no effect on colorectal cancer (Caco‐2) cells.

**TABLE 5:** Cytotoxic properties of lactic acid fermented sour cherry beverages, analyzed on day and after 28 days of storage on MCF‐7.

C (μL/mL)	Cell viability (%)					
	SCN9‐Control	SCN12‐Control	SCN9‐SH10	SCN12‐SH5	SCN12‐SH14	SCN9‐Mix
**Day 0**						
100	101.69 ± 0.99^Ab^	102.26 ± 1.81^Aa^	89.87 ± 3.94^BCb^	93.31 ± 2.23^BCa^	87.64 ± 1.98^Cd^	94.45 ± 0.78^Bc^
50	102.37 ± 1.08^Aab^	103.21 ± 2.62^Aa^	97.91 ± 2.58^ABab^	99.25 ± 2.12^Aa^	93.20 ± 1.39^Bc^	98.64 ± 1.56^Ab^
25	103.81 ± 1.10^Aab^	104.19 ± 1.46^Aa^	100.95 ± 2.16^Aba^	99.69 ± 3.29^Aba^	97.40 ± 0.27^Bb^	100.10 ± 1.20^ABab^
12.5	103.93 ± 0.68^Aab^	105.62 ± 2.93^Aa^	101.83 ± 4.92^Aa^	99.47 ± 0.67^Aa^	99.84 ± 0.56^Aab^	101.20 ± 0.65^Aab^
6.25	104.35 ± 0.95^Aab^	106.00 ± 2.23^Aa^	101.11 ± 2.34^Aa^	101.44 ± 4.00^Aa^	100.73 ± 0.88^Aab^	101.64 ± 0.73^Aa^
3.125	104.47 ± 1.56^Aab^	106.73 ± 4.11^Aa^	102.48 ± 4.72^Aa^	101.22 ± 7.24^Aa^	101.21 ± 2.34^Aa^	102.25 ± 0.92^Aa^
1.562	104.80 ± 0.83^Aa^	107.12 ± 1.68^Aa^	102.15 ± 4.42^Aa^	102.44 ± 5.97^Aa^	102.46 ± 0.60^Aa^	102.52 ± 1.36^Aa^
**Day 28**						
100	104.00 ± 1.64^ABb^	105.97 ± 3.98^Aa^	100.57 ± 0.27^Bc^	100.60 ± 0.15^Bd^	100.58 ± 0.15^Bc^	100.35 ± 0.11^Bc^
50	105.92 ± 0.83^Aab^	106.84 ± 1.19^Aa^	102.03 ± 0.67^Bbc^	102.44 ± 0.52^Bcd^	102.60 ± 0.15^Babc^	102.24 ± 0.50^Bbc^
25	106.27 ± 0.53^Aab^	107.00 ± 1.00^Aa^	103.09 ± 0.37^Babc^	102.28 ± 0.60^Bbcd^	102.12 ± 0.38^Bbc^	102.08 ± 0.29^Bbc^
12.5	106.37 ± 0.79^Aab^	107.12 ± 2.32^Aa^	103.55 ± 0.94^Aabc^	103.31 ± 1.25^Abcd^	103.48 ± 1.16^Aabc^	103.80 ± 1.80^Aabc^
6.25	106.47 ± 1.34^Aab^	107.18 ± 1.03^Aa^	103.96 ± 1.69^Aab^	104.14 ± 1.56^Aabc^	104.39 ± 2.19^Aab^	104.74 ± 2.77^Aab^
3.125	107.59 ± 0.92^Aba^	108.76 ± 0.92^Aa^	105.31 ± 1.46^Bab^	105.43 ± 1.79^ABab^	104.52 ± 1.13^Bab^	106.48 ± 1.08^ABa^
1.562	107.63 ± 0.92^Aa^	108.96 ± 2.15^Aa^	105.61 ± 1.89^Aa^	106.58 ± 0.98^Aa^	105.88 ± 2.13^Aa^	106.40 ± 0.53^Aa^

*Note:* Different lowercase letters in the tables indicate significant differences among sour cherry beverage formulations, whereas different uppercase letters indicate significant differences across storage time (two‐way ANOVA, Tukey's multiple comparison test, *p* < 0.05).

Abbreviations: C, Concentration; SCN, Sour cherry nectar; SCN12‐SH14, SCN containing 12% sucrose inoculated with *Lactiplantibacillus pentosus* SH14; SCN12‐SH5, SCN containing 12% sucrose inoculated with *Lactiplantibacillus plantarum* SH5; SCN9‐Control and SCN12‐Control, Noninoculated SCN samples containing 9% and 12% sucrose, respectively; SCN9‐Mix, SCN containing 9% sucrose inoculated with a mixed culture of SH5, SH10, and SH14; SCN9‐SH10, SCN containing 9% sucrose inoculated with *Limosilactobacillus fermentum* SH10.

### 3.14 In Vitro Bioaccessibility

**FIGURE 3:**
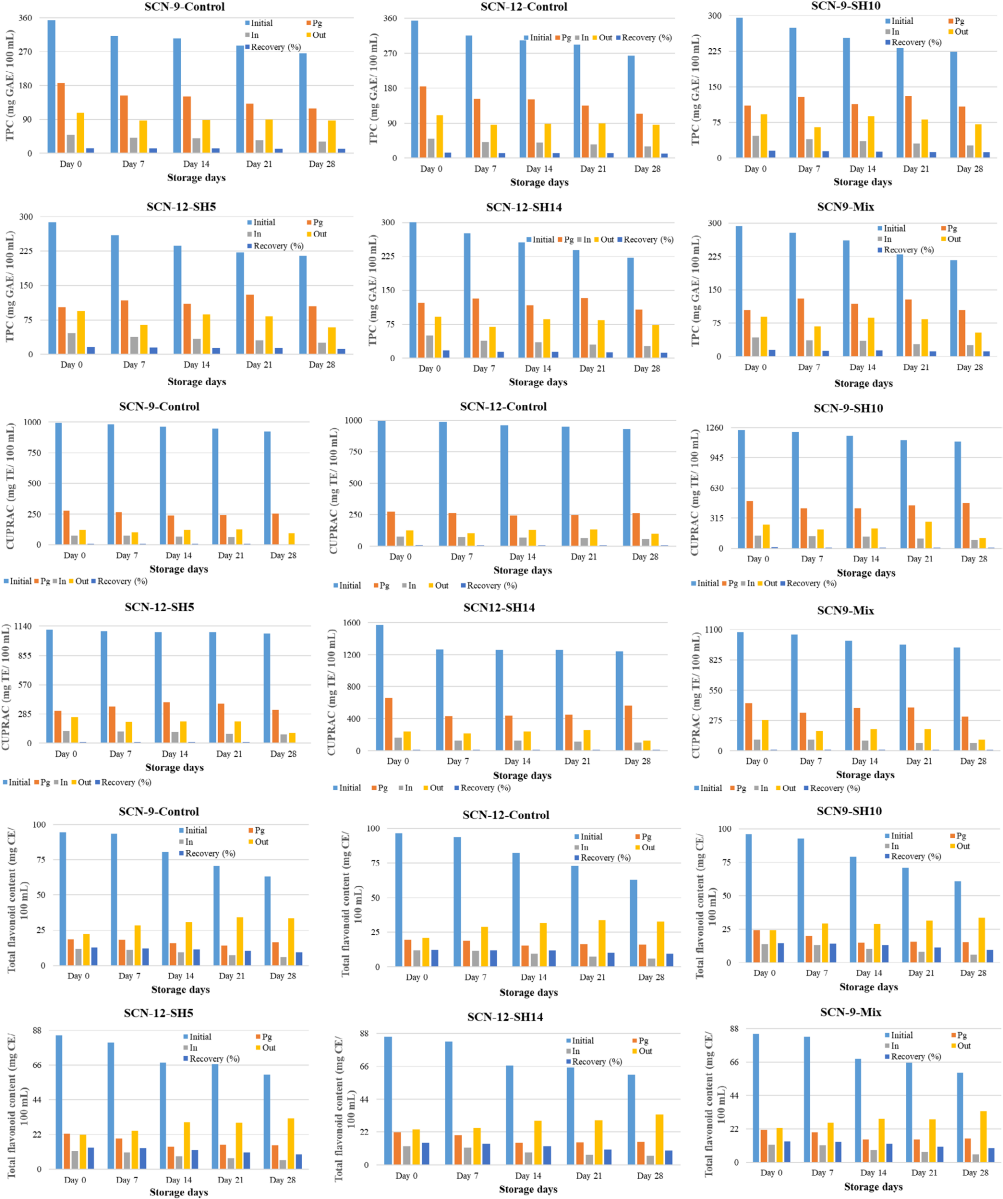
TPC, CUPRAC, and total flavonoid content of lactic acid fermented sour cherry beverages during 28 days of storage, evaluated at 7‐day intervals throughout the gastrointestinal digestion phases. In, intestinal phase (inside dialysis membrane/bioaccessible fraction); Initial, value before in vitro digestion; Out, Intestinal phase (outside dialysis membrane/nonbioaccessible fraction); Pg, Post‐gastric phase; Recovery (%), Percentage of the compound retained after digestion relative to the initial value; SCN, Sour cherry nectar; SCN12‐SH14, SCN containing 12% sucrose inoculated with *Lactiplantibacillus pentosus* SH14; SCN12‐SH5, SCN containing 12% sucrose inoculated with *Lactiplantibacillus plantarum* SH5; SCN9‐Control and SCN12‐Control, Noninoculated SCN samples containing 9% and 12% sucrose, respectively; SCN9‐Mix, SCN containing 9% sucrose inoculated with a mixed culture of SH5, SH10, and SH14; SCN9‐SH10, SCN containing 9% sucrose inoculated with *Limosilactobacillus fermentum* SH10.

The in vitro bioaccessibility values of lacto‐fermented sour cherry–based beverages under simulated gastrointestinal conditions during storage (Days 0, 7, 14, 21, and 28) are presented in Figure 3. A marked reduction in TPC was observed following gastric digestion (*P*_g_). In the subsequent intestinal phase (IN), TPC values indicated that fermentation did not significantly enhance the serum availability of phenolic compounds, as the IN fractions of fermented beverages were generally lower than those of the nonfermented control samples. In contrast, the OUT fraction, representing the nonabsorbable portion of intestinal digestion, consistently displayed higher TPC levels than the IN fraction across all samples, suggesting that a considerable proportion of phenolic compounds withstand intestinal digestion but remain unavailable for serum absorption. Overall, TPC values of the control samples were higher than those of the fermented beverages across the initial, *P*_g_, IN, and OUT phases. However, when considering percentage recovery values, which reflect bioaccessibility, lacto‐fermented samples demonstrated superior bioaccessibility during digestion compared to controls. These findings align with previous reports. Méndez‐Galarraga et al. (2025) emphasized that, despite a reduction in TPC, fermentation frequently improves phenolic bioaccessibility due to probiotic‐induced effects that facilitate their release during digestion. This enhancement has been attributed to the depolymerization of long‐chain phenolic compounds into shorter derivatives with greater intestinal permeability (Ozkan et al. 2022), as well as the increased hydrolysis of polyphenol–protein and polyphenol–carbohydrate complexes in fermented samples by digestive enzymes (Méndez‐Galarraga et al. 2025). Similar patterns were observed for total antioxidant capacity and flavonoid content. Consistent with these results, Dogan et al. (2021) reported increased TPC and recovery (%) in lacto‐fermented vegetable beverages, while Ozkan et al. (2022) demonstrated superior antioxidant activity and recovery rates in fermented thyme leaf extracts compared to nonfermented controls. Méndez‐Galarraga et al. (2025) further suggested that this effect arises from metabolic alterations in the food matrix induced by probiotic strains, which restructure the matrix to facilitate phenolic extraction and release during digestion. Although certain phenolics may be metabolized by bacteria, such restructuring enhances their presence in intestinal digests and improves overall bioaccessibility.

When the percentage of recovery values are examined in detail, clear strain‐dependent improvements in phenolic bioaccessibility are evident. On Day 0, the recovery of phenolics in control samples was approximately 14.05%–14.08%, whereas fermented samples exhibited higher values ranging from 14.58% to 16.45%. The highest bioaccessibility was observed in SCN‐12‐LPN (16.45%), followed by SCN‐9‐LF (16.15%) and SCN‐12‐LP (15.89%). A similar trend was maintained on Day 7, where controls showed ~13.1% recovery, while fermented samples ranged between 14.08% and 14.82%. Although the difference gradually narrowed during storage, fermented samples consistently maintained higher recovery percentages than controls even on Day 28 (11.81%–11.84% vs. 11.46%–11.63%). These results clearly demonstrate that fermentation, particularly with SH14 and SH10 strains, enhanced the bioaccessibility of phenolic compounds during simulated digestion. This quantitative evidence explains why the increase in bioaccessibility is not immediately apparent from TPC values alone but becomes clear when % recovery is considered.

In addition, Zhao et al. (2025) highlighted that the bioaccessibility of phenolics is strongly influenced by their enzymatic transformation, and variations in TPC across different substrates reflect strain‐specific capacities, underscoring the importance of appropriate LAB strain selection. Furthermore, it has been reported that hydroxycinnamic acids often occur as tartaric acid esters and diesters, including caffeoyltartaric, p‐coumaroyltartaric, and feruloyltartaric acids, which may be hydrolyzed during processing or storage to release their corresponding free acids, such as caffeic, p‐coumaric, and ferulic acids. In addition, the enhancement in phenolic bioaccessibility observed in this study can be associated with the well‐documented enzymatic profiles of the LAB strains used. *Lactiplantibacillus plantarum, Limosilactobacillus fermentum*, and *Lactiplantibacillus pentosus* are known to possess β‐glucosidase, esterase (particularly feruloyl esterase), and phenolic acid decarboxylase activities (Paventi et al. 2025; Tian et al. 2026; Zhang et al. 2024). These enzymes play a crucial role in the biotransformation of phenolic compounds during fermentation (Zhang et al. 2024). β‐Glucosidase facilitates the hydrolysis of glycosidically bound phenolics into their free and more bioaccessible forms (Xie et al. 2026), while esterase activity promotes the hydrolysis of hydroxycinnamic acid esters such as caffeoyltartaric, p‐coumaroyltartaric, and feruloyltartaric acids, leading to the release of their corresponding free acids (Andreasen et al. 2001; Schär 2016). Additionally, phenolic acid decarboxylase contributes to structural modification of phenolic acids, which may further influence their antioxidant potential and bioavailability (Kumar et al. 2025; Shahidi and Peng 2018). Therefore, the strain‐specific enzymatic capacity of these LAB strains directly explains the variations observed in TPC and phenolic profiles across different substrates, highlighting that the increase in antioxidant availability is not merely substrate‐dependent but strongly linked to the metabolic and enzymatic characteristics of the selected LAB strains. In line with this, Chen et al. (2023) observed that in lactic acid–fermented sour cherry–based beverages, compounds such as chlorogenic acid, syringic acid, ferulic acid, (+)‐catechin, and rutin were present at lower concentrations than in control samples, suggesting their involvement in LAB‐mediated metabolic processes or their conversion into alternative metabolites. Moreover, Erol et al. (2024) reported that phenolic compounds, particularly anthocyanins, exhibit high sensitivity to neutral or alkaline conditions, with their stability being greater under acidic pH and influenced by their specific chemical structures.

## 4 Conclusion

This study demonstrated the potential of developing lacto‐fermented sour cherry‐based beverages with probiotic functionality. Among the tested strains, *Lactiplantibacillus plantarum* SH5, *Limosilactobacillus fermentum* SH10, and *Lactiplantibacillus pentosus* SH14 were identified as optimal starter cultures. The results showed that fermentation resulted in beverages with reduced caloric content, fluctuating phenolic composition, enhanced antioxidant activity, improved in vitro bioaccessibility of bioactive compounds, and concentration‐dependent cytotoxic effects against MCF‐7 breast cancer cells. Although the tested LAB strains adapted successfully to the sour cherry matrix, microbial growth during fermentation was limited, and viable counts declined rapidly during storage. The beverages retained LAB populations above 10^6^ CFU/mL for up to 28 days; however, a shorter shelf life of approximately 14 days at 4°C is recommended to ensure probiotic functionality. Physicochemical analyses revealed slight but significant reductions in °Brix and dry matter content, accompanied by increased turbidity during storage. Fermentation caused significant and perceptible color changes compared to controls, while total phenolic content decreased progressively during fermentation and storage. Nevertheless, antioxidant capacity was consistently higher in fermented samples. Phenolic profiling showed catechin, chlorogenic acid, and myricetin as predominant compounds, with most phenolics decreasing during storage. Antibacterial activity was only observed in the SCN9‐Mix and control samples against *Bacillus cereus* . Cytotoxicity assays indicated relatively low inhibitory effects toward MCF‐7 breast cancer cells, which further diminished during storage. Furthermore, although TPC values of controls were initially higher than those of fermented beverages, the superior percentage recovery values of lacto‐fermented samples highlighted improved bioaccessibility during in vitro digestion. Overall, these findings suggest that sour cherry juice can effectively support the growth and metabolic activity of some LAB strains during fermentation; however, the maintenance of high probiotic viability during extended storage is clearly strain‐dependent. Future studies should focus on evaluating the sensory attributes, particularly the effects of selected LAB strains on the flavor and aroma profile of the beverages, to ensure consumer acceptability alongside functional benefits.

## Author Contributions

**Perihan Kubra Akman:** conceptualization, investigation, writing – original draft, methodology, formal analysis, project administration, data curation, resources, writing – review and editing. **Gulsum Ucak‐Ozkaya:** investigation, formal analysis, data curation, validation. **Gozde Kutlu:** investigation, writing – original draft, writing – review and editing, visualization. **Fatih Tornuk:** writing – review and editing, project administration, data curation, supervision, resources. **Hasan Yetim:** project administration, data curation, supervision, resources.

## Funding

This study was supported by The Scientific and Technological Research Council of Türkiye (TUBITAK) under Program Code: 1005, Project No: 120O203. Ethical approval was not required for this study.

## Conflicts of Interest

The authors declare no conflicts of interest.

## Data Availability

Data will be made available on request.
